# New estimate of chemical weathering rate in Xijiang River Basin based on multi-model

**DOI:** 10.1038/s41598-021-84602-1

**Published:** 2021-03-11

**Authors:** Yong Zhang, Shi Yu, Shiyi He, Pingan Sun, Fu Wu, Zhenyu Liu, Haiyan Zhu, Xiao Li, Peng Zeng

**Affiliations:** 1 The Guangxi Zhuang Autonomous Region geological environment monitoring station, Nanning, 530029 China; 2 Institute of Karst Geology CAGS/Key Laboratory of Karst Dynamics, MNR & GZAR, Guilin, 541004 China; 3 Guangxi Branch of China National Geological Exploration Center of Building Material Industry, Guilin, 541004 China; 4 College of Geography and Environmental Science, Northwest Normal University, Lanzhou, 730070 China; 5 College of Environmental Science and Engineering, Guilin University of Technology, Guilin, 514006 China

**Keywords:** Geochemistry, Hydrology

## Abstract

Hydrochemistry and Sr isotope compositions were measured in water samples collected during high- and low-water periods from the main stream and tributaries of the Xijiang River Basin in southern China. The primary weathering end-members were analyzed and calculated using the multi-model combination and classic hydrogeochemical method. During the high-water period, structural factors were found to be the main factors controlling chemical weathering in the basin, whereas anthropogenic activity and other random factors had a negligible influence. During the low-water period, both structural and random factors controlled chemical weathering. Through path-model and semi-variance analyses, we determined and quantified the relationship between the main weathering sources, whose results were stable; this is consistent with the inversion model. The total dissolved substances were mainly derived from carbonate weathering, which was approximately 76% (0–96%) while silicate weathering accounted for only 14% (5–19%). The inversion model results showed that the optimum silicate weathering rate was 7.264–35.551 × 10^3^ mol/km^2^/year, where carbonic acid was the main factor that induces weathering. The CO_2_ flux consumed by rock weathering in the basin during the study period was 150.69 × 10^9^ mol/year, while the CO_2_ flux consumed by carbonic acid weathering of carbonate (CCW) and silicate rocks (CSW) was 144.47 and 29.45 × 10^9^ mol/year, respectively. The CO_2_ flux produced by H_2_SO_4_ weathered carbonate (SCW) was 23.23 × 10^9^ mol/year.

## Introduction

As a critical component of the global water cycle, rivers are the main channel that connect the land ecosystem and ocean, two major active carbon pools. The output of dissolved inorganic carbon (DIC; mainly HCO_3_^−^) in rivers reflects the intensity of atmospheric CO_2_ consumption by chemical weathering in river basins, which is generally considered a natural carbon sink process. Rivers transfer approximately 0.43 Pg of inorganic carbon to the ocean every year^1^ such that this is a main component of the missing carbon sink, as well as an important topic in global carbon cycle research^2–6^. Revealing the effect of chemical weathering on the carbon cycle and quantitatively determining the absorption flux of CO_2_ by rock weathering are vital steps for determining the mechanism(s) of the long-term carbon cycle and climate change.

In the long-term global carbon cycle, the net consumption of atmospheric CO_2_ caused by the chemical weathering of silicates plays an important role^7^. As this consumed CO_2_ is partially fixed in sediments in lakes and oceans, it cannot return to the atmosphere on a short time scale (silicates: millions of years, carbonates: decades to thousands of years)^8–11^. Previous studies have suggested that the carbon sink of silicate chemical weathering controls climate change over long time scales^12^.

The weathering of carbonates can also rapidly consume atmospheric CO_2_. However, this CO_2_ returns to the atmosphere through the deposition of carbonate minerals. The Fifth Intergovernmental Panel on Climate Change (IPCC) report confirmed that the inorganic carbon flux from the chemical weathering of carbonate rocks is a carbon sink on the century-to-millennium time scale^13–15^. This carbon sink is considered unstable, which has resulted in disputes regarding the proposal of carbonate weathering as a carbon sink in the global carbon budget^16^.

In the chemical weathering process, carbonate weathering with sulfuric acid participation can also produce HCO_3_^−^. However, due to the longer retention time of SO_4_^2−^ in seawater (8.7 Ma), the retention time of HCO_3_^−^ is only 0.083 Ma^17^. With the precipitation of carbonate minerals, half of the HCO_3_^−^ is re-released into the atmosphere in the form of CO_2_. Therefore, from this perspective, the participation of H_2_SO_4_ in carbonate weathering is essentially a net release of atmospheric CO_2_ process. When calculating the carbon flux, this part of the HCO_3_^−^ produced by the H_2_SO_4_ weathered carbonate rock must be deducted^18^. While the research on the chemical weathering intensity of the marine sedimentary carbonate rock formation is relatively few^19^, the actual scenario may be more complex. In addition, chemical weathering is also affected by geomorphological units^20,21^. In mountainous environments, different slope orientations have different weathering rates due to the differences in humidity^22^; glaciers have strong physical and chemical weathering^23^. In previous studies, a variety of isotopes have been used to prove that the chemical weathering input of glacial melt water cannot be ignored and has important global carbon cycle significance^24,25^. Human activities are also an important factor affecting rock weathering, especially the karst environment which is fragile^26,27^ and prone to quick penetration from pollutants such as domestic sewage and agricultural fertilizers^28,29^. This results in the high mobility of nitrogen^30^, which directly leads to the action of ammonia nitrogen in the soil layer to control the hydrogeochemical process in the hydrochemical evolution^31,32^.

For an in-depth study of the carbon sink due to rock weathering and its controlling mechanism, we must more accurately evaluate the contribution of each end-member, especially in rivers. This places increased importance on the calculation method, assessment technique, and constructed model for the carbon sink effect. Accurately assessing the total flux and proportion of each end-member is especially challenging.

Located in southwestern China, the Xijiang River Basin has a typical subtropical monsoon climate, with rain and heat in the same period of the year. Carbonate is widely distributed in this area, making it a particularly important area to study rock chemical weathering. Several previous studies have examined chemical weathering processes in the region based on spatial and temporal sampling campaigns^33,34^. Other studies have analyzed the riverine ion concentrations, chemical weathering characteristics under climate control, and chemical weathering carbon flux at different spatial and temporal scales^35–38^.

These studies focused on the influences of exogenous acid, lithological control, water cycle, and other factors associated with the chemical weathering process. However, these studies did not sufficiently examine the contribution of different carbonate end-members (dolomite and limestone) and the impact of anthropogenic activity. In this study, water samples were systematically collected from the main stream and tributaries of the Xijiang River Basin during high- and low-water periods. A multi-model combination and classic hydrogeochemical method was adopted to analyze the hydrochemistry and Sr isotope compositions of the river water as follows. (1) A semi-variance model was used to discuss the variability of Sr and its isotopic composition to reduce error when estimating rock weathering due to anthropogenic activity. (2) A path model for Sr and its isotopes was used to refine the contribution of each end-member to river chemical weathering. (3) The total CO_2_ flux and contribution from each end-member were calculated with the inversion model. (4) Based on the above results, we discussed the application of the model to estimate natural chemical weathering processes and anthropogenic influences.

## Natural setting of the Xijiang River Basin

### Hydrological situation

Xijiang is the main river of the Pearl River Basin, originating from Maxiong Mountain in Zhanyi County, Yunnan Province. The Xijiang flows through Guizhou, Guangxi, and into Guangdong, merging with Beijiang at Xianjiao to the west of Guangdong. The total length of the Xijiang is 2075 km, with an average gradient of 0.58% and a drainage area of 353,100 km^2^. The main stem of the Xijiang is divided into five sections from top to bottom: Nanpan River, Hongshui River, Qianjiang, Minjiang, and Xijiang. The Xijiang River Basin is characterized by a typical subtropical monsoon climate zone. There is significant variation in the annual runoff, with an average annual runoff of approximately 230 billion m^3^. The high water period occurs from April to September, accounting for approximately 72–88% of the annual runoff.

### Geological setting

Xijiang River is the western tributary of the Pearl River, which originates in the Yunnan–Guizhou Plateau and flows into the South China Sea through Yunnan, Guizhou, Guangxi, and Guangdong Provinces. The stratigraphic lithology of the Xijiang River Basin is complex, ranging from Cambrian metamorphic rocks to Quaternary sedimentary rocks (both mainly sedimentary and magmatic rocks). Carbonates (mainly limestone) are widely distributed in the basin. Their outcrop area accounts for approximately 44% of the basin surface, mainly in the upper and middle reaches. Karst is an important factor affecting the natural environment of the basin.

#### Sedimentary rocks

Exposed sedimentary rocks in the basin range from pre-Sinian to Quaternary, where the most developed are Devonian, Carboniferous, Permian, and Triassic.

*Pre-Cambrian* The highest old stratum discovered in the Xijiang River Basin is the Sibao Group of Mesoproterozoic age, mainly composed of metamorphic shale, which occurs in the southeastern Guizhou-northern Guizhou area. The Sinian is widely distributed in the basin, located at the top of the upper Paleoproterozoic and underlies the Cambrian in the lower part of the early Paleozoic. In general, Pre-Cambrian strata are pre-marine deposits, mainly consisting of shallow metamorphic clastic rocks with a small amount of carbonate.

*Cambrian to Triassic* Most sedimentary environments in this area are shallow sea, including the clastic rock-carbonate formation of neritic facies from the early Paleozoic (mainly clastic rock) and carbonate-clastic rock formation of neritic facies from the late Paleozoic. The Permian was characterized by a period of continental-marine interaction, dominated by carbonate with clastic rocks and coal seams, which were the main coal-bearing strata in the basin. The Triassic lithology is complex. Early Paleozoic strata are widely distributed in the basin while late Paleozoic and Triassic strata are the most developed. Carbonate rocks, the main karst strata in the basin, are often exposed in patches.

*Jurassic to Cenozoic* Since the late Triassic, this region has entered a continental sedimentary period. Sediments are mainly composed of clastic rocks, where sandy shale and pyroclastic rock dominate the Jurassic, with thin coal seams.

#### Magmatic rocks

Magmatic rocks are predominantly distributed in the eastern part of the basin, mainly in eastern Guangxi and Guangdong, while only sporadically in other areas. The majority of these magmatic rocks are granitoids. The most widely distributed magmatic rocks in the Xijiang River Basin are Yanshanian (i.e., granite-dominated intermediate-acid intrusive rocks and intermediate-acid extrusive rocks), followed by the Indosinian and Caledonian. Yanshanian rocks are concentrated in southeastern Guangxi and Guangdong, Mesozoic rocks are mainly in western and southeastern Guangxi, and Hercynian rocks are distributed in the Zhenfeng–Luodian area along the Beipanjiang River in the Hongshuihe River Basin.

### Climate, vegetation, and population

From June 2014 to January 2015, the Xijiang River Basin is located in a subtropical region with a mild climate (average annual temperature: 14–22 °C) from. The average annual temperature of the upper reaches in the Nanpan River at Kaiyuan is 19.8 °C while that at Luodian along the Beipan River is 19.6 °C. These temperatures are relatively higher on the Yunnan–Guizhou Plateau. The average annual temperature of Guangxi in the middle of the basin ranges from 18.8 to 22.1 °C while the mean perennial temperature in the delta region of the lower basin ranges from 20.3 to 21.8 °C.

The study area has abundant rainfall, with an average annual value of 1470 mm that decreases from east to west. The average rainfall in Mengzi County (southeastern Yunnan) is the lowest (720 mm). Precipitation in the basin originates from the South China Sea, West Pacific, and Bay of Bengal. The southeast monsoon from March to May imports vapor from the West Pacific, affecting areas east of 105°E. The southwest monsoon prevails from May to August, transporting vapor from the Bay of Bengal and South China Sea to areas west of 110°E.

The average annual evaporation in the basin ranges from 900 to 1600 mm, which is generally low in the north and high in the southeast from June 2014 to January 2015. The average monthly evaporation in April or May is the highest in the western Yunnan–Guizhou Plateau region, whereas the evaporation in July is the highest in Guangdong and Guangxi provinces.

The main types of vegetation in the upper reaches of the Xijiang River Basin are evergreen oak and pine forests in the middle and subtropical region. The lower reaches of the North–South Panjiang River and hilly areas of the Red Creek Valley are covered by rainforests at the northern margin of the tropics and vegetation in the southern subtropics below 800 m. The vegetation in the Guangxi Basin in the middle reaches of the Xijiang River is mainly subtropical evergreen broad-leaved forest and northern subtropical evergreen seasonal rainforest. The average population density is low (199 people per km^2^).

## Sampling and analysis methods

### Sampling and analytical procedures

**Figure 1:**
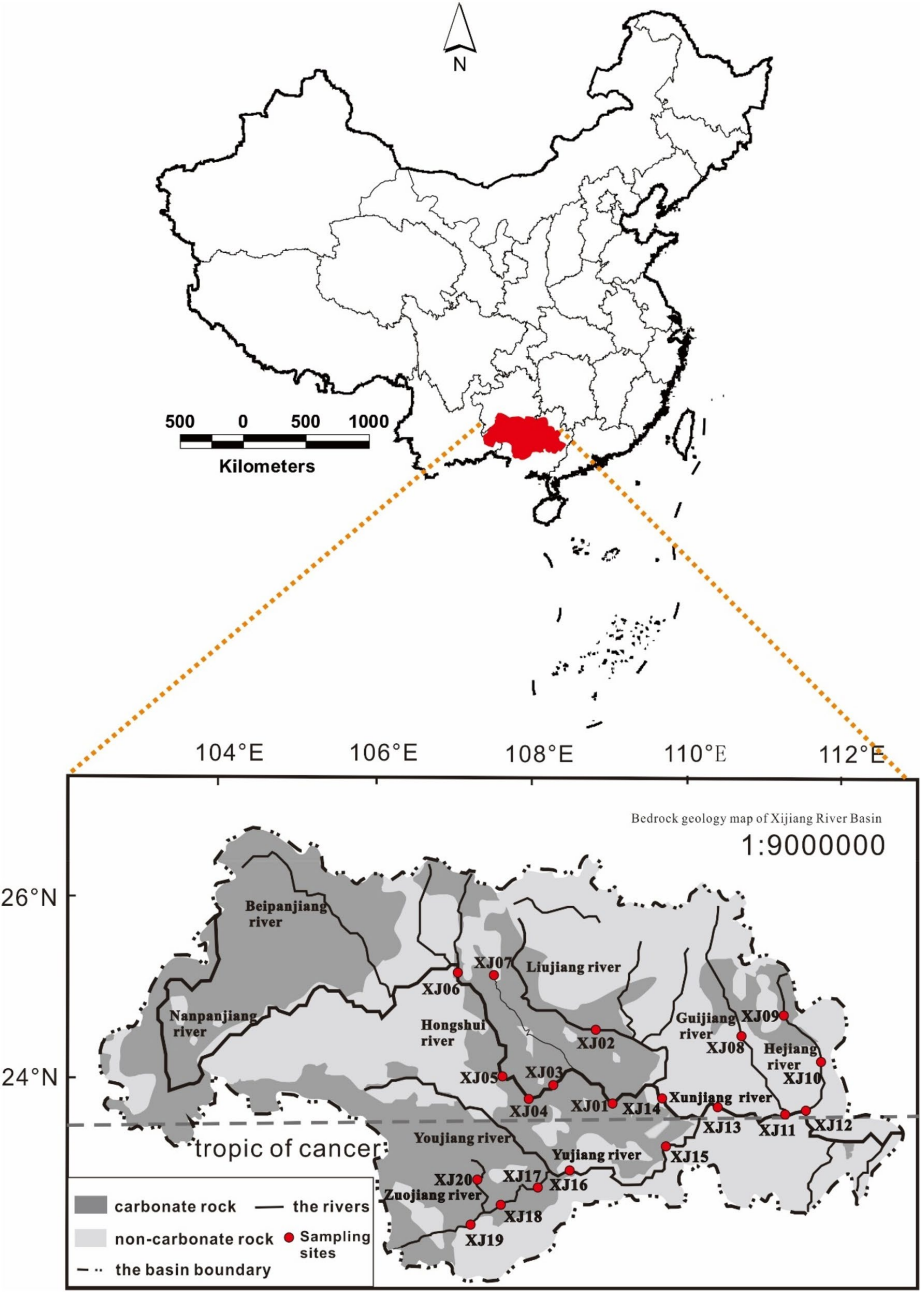
The hydrogeology and sampling point distribution in the Xijiang River Basin (this figure updated from Yu et al.^38^).

The high water period of the Xijiang River Basin is generally from April to September, and the low water period is generally from January to March and October to December. The collection and monitoring of Xijiang samples are divided into low and high water periods. For the Xijiang River basin, the months with more precipitation are from May to August, and the months with less precipitation are January, February, and December. To make sampling and monitoring more representative, sunny or cloudy days with relatively stable meteorological factors were selected. In June 2014 (representing the high water period) and January 2015 (representing the low water period), water sampling was carried out at the main stream of the Xijiang River Basin, first-level tributaries, and second-level tributaries (Fig. 1). A total of 20 sampling sites were sampled during high- and low-water periods. During the same months, the rainfall in the basin was collected and analyzed. Two rainfall monitoring stations were set up in densely populated areas, and one rainfall monitoring station was set in suburban areas with less human activities. The annual rainfall monitoring was conducted in 2016. A total of 240 rainfall samples were collected, including 174 rainfall samples in densely populated areas and 66 rainfall samples in suburbs. The sample results were used for model calculation and model result characterization.

River water was collected approximately 10 cm below the surface. After rinsing the sampling bottle with water 3–4 times, the water sample was collected, filtered through a 0.45 μm cellulose acetate lipid membrane, and stored into a 50 mL polyethylene bottle. The bottle was then immediately placed in a portable ice bag, transported to the storage room within 12 h, and refrigerated at 4 °C. For cation analysis, the filtered water samples were acidified to a pH of less than 2 using ultra-purified HNO_3_.

The water pH, temperature, dissolved oxygen, and electrical conductivity were measured using an ODEON multi-parameter water quality analyzer (Ponsel, France), with respective accuracies of 0.01, 0.1 °C, 0.01 mg/L, and 1 μs/cm. The temperature was automatically compensated. The HCO_3_^−^ and Ca^2+^ concentrations in the water were measured on-site with alkalinity and hardness kits (Merck, Germany), with accuracies of 0.1 mmol/L and 2 mg/L, respectively.

The Cl^−^, NO_3_^−^, and SO_4_^2−^ anions were analyzed via an ion chromatograph (861 Advanced Compact IC Metrohm, Switzerland). The K^+^, Na^+^, Ca^2+^, and Mg^2+^ cations were determined via inductively coupled plasma optical emission spectroscopy (ICP-OES, IRIS Intrepid II XSP, Thermo Fisher Scientific, U.S.A.). The accuracy of the anion and cation concentrations was 0.01 mg/L, with an analysis error of less than 5%. We determined the SiO_2_ via the silicomolybdic yellow colorimetric method (DZ/t0064.62-1993), with an accuracy of 0.01 mg/L. Water chemistry analyses were performed at the Karst Dynamics Laboratory of the Ministry of Land and Resources/Guangxi at the Institute of Karst Geology, Chinese Academy of Geological Sciences (Guilin, China).

The ^87^Sr/^86^Sr ratios were measured in the isotope laboratory at the Yichang Institute of Geology and Mineral Resources, China Geological Survey. Water samples were dried in a polytetrafluoroethylene sample dissolver, dissolved in an appropriate amount of HCl, and then dried again. The purified liquid was extracted using 1 mol/L HCl, and passed through a Dowex 50 × 8 (200–400) cation exchange column. The Sr was then separated and purified for isotope analysis by mass spectrometry (MAT-263). The NBS987 and GBW04411 standard references were used to monitor the instrument and analytical process, with a standard value for NBS987 of 0.71034 ± 0.00002 (2δ). All sample preparation for isotopic analysis was performed in a super-clean laboratory. The Sr blanks were < 5 × 10^−9^ g.

### Methods

#### Multi-model combination and classic hydrogeochemical method

Inverse models are generally used as a standard method to calculate the chemical weathering flux^39^. This method is based on the assumption that river solutes derive from four end-members: atmospheric sedimentation, evaporite dissolution, and the weathering of carbonates and silicates. Based on the products of different end-members, a chemical weathering mixing model can be constructed using different Na-normalized elemental ratios of the major elements and Sr isotopic ratios. The inversion model has two advantages. (1) The weathered Sr isotope end-members derive from relatively similar sources, which can better reflect the origin of river solutes. (2) Strontium isotopes are relatively stable such that the error in the final calculation results is small. The traditional inversion model, however, is also inadequate in several aspects. First, there are relatively few studies on the influence that anthropogenic activity has on Sr isotopes. Despite their relative stability, Sr isotopes can be slightly affected by certain anthropogenic activities, which, in turn, will affect the calculation results of the inversion model. However, previous studies have found that, although anthropogenic activities have an impact on the Sr isotope ratio, there is no definitive qualitative or quantitative equation, or even specific description, to estimate the influence from anthropogenic activities. Thus, the inversion model is not suitable for calculating chemical weathering fluxes if anthropogenic activities have had a large impact. Second, although the inversion model can sufficiently calculate the contribution of each end-member, the subdivision of carbonate weathering (including limestone and dolomite weathering) has not been clarified. Previous studies generally consider a combination of dolomite and limestone to calculate the proportion of carbonate weathering^40^. Thus, in specific typical karst basins, considering the subdivision of carbonate weathering can allow us to elucidate the mechanism of chemical weathering. Finally, whether the model’s calculation results represent the actual weathering degree is a matter for debate. In general, studies on chemical weathering involve certain limitations with respect to the experimental design and sampling^41^. For example, the measured index may differ between samples collected in the morning and afternoon at the same place, whose underlying cause has not been explored in depth. In large-scale watershed studies, there are restrictions on the sampling frequency and distribution accuracy. The sampling frequency is usually divided roughly into high- and low-water periods, where the number of samples generally does not exceed 100. In addition, the river water index continues to change dynamically while samples only reflect the situation at the time of collection or during that quarter, which does not necessarily reflect the actual situation for the entire basin.

Based on these aspects, we adjusted the analysis and calculation in this study as follows. The semi-variance model was employed to clarify the spatial structure, including the influence of anthropogenic activities. This model was combined with the classic hydrogeochemical method (i.e., to obtain the random and structural factors), path model (i.e., to analyze and verify the relationship between the two types of obtained factors), and inversion model (i.e., to verify the weathering ratio of the end-members). The overall approach, which is termed the “multi-model combination and classic hydrogeochemical weathering,” is an organic framework. In addition, this approach improves the algorithm for calculating the end-members in the inversion model, and further quantitatively estimates the material source separately using the weathering characteristics of limestone and dolomite.

#### Semi-variance analysis

Natural biological processes consist of both random and structural variables. The latter have a certain degree of correlation or continuity in their spatial distribution. For example, Sr and its isotopes have spatial distribution characteristics, where the Sr isotope ratio reflects the geological and lithological characteristics along the course of the river, i.e., essentially regional variables. Structural variables are also susceptible to anthropogenic activities that cannot fully reflect its indicator significance^42^. The study of Sr and its isotopes based on hydrogeochemical analyses does not consider the spatial autocorrelation of the research objectives. Therefore, they cannot provide more detailed information on the degree and scope of aggregation (or randomness)^43,44^.

Geostatistics is a set of theories and methods for analyzing spatially related variables based on the combination of geological and statistical analyses. According to the theory of regionalized variables, as well as using the semi-variance function as the main tool, geostatistics can be used to examine natural phenomena that have both randomness and a spatial distribution structure, thereby fully using available information provided by field investigations to reveal the nature of periodic and non-periodic ecological parameters. This approach effectively avoids systematic errors and can produce more accurate quantitative estimates. The specific formula is as follows:

1 $${\text{r}}\left( {\text{h}} \right) \, = {\text{ E/}}2\left[ {{\text{Z}}\left( {\text{x}} \right) \, {-}{\text{ Z}}\left( {{\text{x }} + {\text{ h}}} \right)} \right]^{2}$$

where h represents the spacing, r(h) represents the semi-variance function, x is the location, and Z(x) is the regionalized variable.

As Sr and its isotopes in water mainly derive from rock weathering and anthropogenic activities, we assume that these factors are dominant. The rock type and water temperature mostly affect rock weathering. The major random factors in this study are anthropogenic factors that cannot be precisely determined. This section focuses on the influence that structural and random factors have on Sr and its isotopes. To clarify the influence that anthropogenic activities have on chemical weathering, we adopted the concept of spatial structure while acknowledging that both structural and random factors influence the material source.

In this study, the spatial structure of Sr and its isotopes in the high- and low-water periods was analyzed with geostatistical methods to understand the spatial distribution dynamics of these indices in the basin, simulate the spatial distribution, and provide spatial information and variation rules for the solutes in the Xijiang River Basin. Traditional software used to solve the semi-variogram model, such as ArcGIS and GS + , can only calculate the semi-variogram model to two digits after the decimal point, which is significantly less than the accuracy required for Sr isotopes. To solve this problem, we used the R Programming Language and gstat package to reduce the calculation error and find a suitable semi-variance model.

#### Path model analysis

The path model analysis, first established by the American scientist Wright in 1918, is a mature method for studying complex systems. It is a statistical method that describes the complex causal relationship among multiple factors and the results, consisting of a path graph and multiple regression equations^46^. When there are multiple influencing factors, the relationship between the factors tends to be complicated, with indirect effects, as well as direct interactions, among the factors. The path model analysis is effective for problems that involve multi-factor dependence, including indirect influences. The path model not only visualizes the quantitative relationship between the independent and dependent variable but also calculates the direct effect that each causal factor has on the outcome and the effect of the indirect factors through the path coefficient. Thus, this approach can fully demonstrate the direct and indirect effects of far-dependent and near-dependent variables on the outcome in a comprehensive network^46^. This is an ideal tool for studying Sr and its isotopes in river water, which form a network system composed of various action factors through compatibility. To quantitatively describe the compatibility network, the path analysis is introduced as a multi-causal modern mathematical tool.

Based on the Partial Least Square (PLS) path model analysis and using the plspm package in the R Language, Sr and its isotopes were considered as a potential variable, taking into account the complexity of the formation lithology in the study area. The water chemistry software PHREEQC2.5.1 was used to calculate the anion gypsum (SIA), calcium carbonate (SIC), dolomite (SID), and gypsum (SIG) saturation indices of the water samples, combined with the conventional chemical components of river samples, to construct a PLS-SEM model of Sr and its isotopes among the structural and random factors. We then used this model to explore the effect of each influencing factor and explain the spatial variation in Sr and its isotopes.

### Derivation and calculation

#### Inversion model calculation and sensitivity analysis

The inversion model configuration was identical to that of Millot et al.^47^. However, as the study area was essentially free of evaporite formations, only three reservoirs were considered, i.e., precipitation, carbonate, and silicate. For this reason, we discuss, in greater detail, the model for the hydrochemical characteristics of the Xijiang River Basin in this section. The inversion model was based on a set of mass budget equations of elemental molar ratios (X = Cl, Ca, Mg, HCO_3_, and Sr) [Eq. (2)] and the Sr isotope ratios of the three reservoirs (precipitation, carbonate, and silicate) [Eq. (3)]:

2 $${\left(\frac X{Na}\right)}_{river}=\sum_i{\left(\frac X{Na}\right)}_ia_{i,Na}$$

3 $${\left(\frac{{87}_{Sr}}{{86}_{Sr}}\right)}_{river}{\left(\frac{Sr}{Na}\right)}_{river}=\sum_i{\left(\frac{{87}_{Sr}}{{86}_{Sr}}\right)}_i{\left(\frac{Sr}{Na}\right)}_ia_{i,Na},$$

where subscript *i* represents the three reservoirs, α_i,Na_ is the mixing proportion of Na for each reservoir, and ∑α_i,Na_ = 1. To maintain mass conservation during weathering, K^+^ and SO_4_^2−^ were eliminated from the model as they are susceptible to biological activities. We used the Na-normalized ratios and isotopic compositions to remove the effects of discharge and evaporation^49^.

To further reduce the error, Eqs. (2) and (3) were weighted by the analysis errors of the elemental and ^87^Sr/^86^Sr ratios, which was solved by the commercial version of the 1stopt 4.0 software. In general, the (X/Na)_*i*_ of a set of end-member reservoirs is used to iteratively solve the sum of α_i,Na_ for the three end-members in each sample, as well as to find the (X/Na)_*i*_ ratio for the other end-members. In this study, a total of 40 samples (20 in the high-water period and 20 in the low-water period) were used. Thus, we solved for 140 model parameters (3*i* × 40α_i, Na_ + 20(X/Na)_*i*_) using 280 equations (6 types × 40 samples for a sum of 240 mass balance equations and 40 constraint equations) by successive iterations. After we obtained the best α_rain_, _Na_, α_sil_, _Na_, and α_carb, Na_ values for each sample and the reservoir constraints (X/Na)_*i*_, these a posteriori values were used to calculate other elemental fractions, such as α_rain, Ca_, α_sil, Ca_, and α_carb, Ca_ (α_sil, Cation_ is the sum coefficient of the four cations obtained from silicate).

For end-member selection, instead of adopting a set of end-members that can satisfy multiple river systems, a maximum number of end-members was selected to adapt to the lithological variation across the study area. According to previous studies^39^, the characteristics of the end-members in each study area were examined in detail to obtain the fine-tuned range for the end-members of the river system in the Xijiang River Basin. The fine-tuned range was in the form of a molar ratio to Na, where the model iterated the correlation operation. The rain end-member was simulated using local precipitation data and methods similar to those in other study areas. Taking into account the characteristics of the stratigraphic lithology, local population, and atmospheric precipitation, we used precipitation data from Guilin for the rain end-member. Specifically, three monitoring stations in Guilin from 2016 were selected to represent different types of areas: two urban monitoring sites (Guilin Environmental Monitoring Station and Longyin Road Primary School) and one suburban site (Guilin University of Electronic Science and Technology Yaoshan Campus). A total of 240 precipitation samples from the three monitoring sites were analyzed from January 2016 to December 2017. The best rain end-members were calculated and iterated to obtain the corresponding parameters. For the carbonate end-members, there is essentially no evaporite formation in the study area with a relatively simple material source. Therefore, we assumed that the carbonate rocks have identical properties as the source of monolithologic streams.

The ^87^Sr/^86^Sr ratio of carbonate in the study area was 0.70779, which was within the range of carbonate on the global scale (0.706–0.709). Furthermore, the Sr content was approximately 100 ppm. Thus, we assigned a slightly radiogenic and high-[Sr] value for the Xijiang River Basin. At the same time, to refine the carbonate weathering end-member, the path model was used to assign parameters to the relationship between the weathering strength of dolomite and limestone and characteristic ratios of both. For the silicate end-member, the silicate composition of the Xijiang River Basin has a higher Mg/Na ratio while granite in the study area has a less radiogenic ^87^Sr/^86^Sr ratio. Therefore, we based the rain end-member on matching seasonal and local precipitation while the ranges in carbonate and silicate end-members were adopted from Moon et al.^41^, as listed in Table 1.

**Table 1:** Parameters of each model end-member.

End member	Ca/Na	Mg/Na	HCO_3_/Na	Cl/Na	1000* Sr/Na	^87^Sr/^86^Sr
**Rain**						
High water period	3.83	1.08	23.14	1.41	16.51	0.709^a^
Low-water period	1.66	0.30	13.58	0.61	9.69	0.709^a^
Carbonate^a^	30–70	12–28	60–140	0.001	50–100	0.707–0.709
Silicate^a^	0.01–0.56	0–0.68	1–3	0.001	1–175	0.708–0.910

^a^Carbonate and silicate parameters from Millot et al.^47^, with precipitation ^87^Sr/^86^Sr ratios from Pu et al.^48^.

The elemental concentrations are the first uncertainty factor that affects model results. According to the normalized inorganic charge balance (NICB) conservative estimate, the mean NICB of the river water samples during the high-water period was 0.4% while the low-water period was 3%. Only three of the 40 samples had NICB values exceeding 10%, where most values were less than 5%. However, the error for the ^87^Sr/^86^Sr ratio was 0.00002. To reduce this error, we weighted the equation by the analysis error (i.e., the basic elemental concentration was 10%, with a ^87^Sr/^86^Sr ratio of 0.00002).

To evaluate the sensitivity of the inverse model to the rain end-member, we tested four scenarios: (1) effects of the ocean, (2) high-water period, (3) low-water period, and (4) annual average (Table 2).

**Table 2:** Inverse model sensitivity tests.

Scenarios	Types	Ca^2+^/Na^+^	Mg^2+^/Na^+^	HCO_3_^−^/Na^+^	1000 × Sr/Na^+^	Cl^−^/Na^+^	^87^Sr/^86^Sr
1	Marine Aerosol^a^	0.022	0.12	0.004	0.19	1.16	0.709
2	High water period	3.83	1.08	23.14	16.51	1.41	0.709
3	Low-water period	1.66	0.30	13.58	9.69	0.61	0.709
4	Annual average	1.75	0.62	10.86	7.74	1.08	0.709
5	Silicate end-member^b^						0.720–0.80
6	Silicate end-member^c^						0.708–0.910

^a,b^Millot et al.^47^^.^

^c^Moon et al.^40^.

During the 12-month sampling period, the main elemental components in rainwater collected at Guilin were averaged, using the corresponding Sr concentrations and isotope ratios. The seasonally matched local rain composition was used in our final optimized run. The reasons for this are as follows. (1) Although river water, to a certain extent, in the study area was affected by marine sports, “Results” section states that the main factors affecting the spatial variation of Sr and its isotopes were structural factors. If we simply consider the impact that the rain end-member has on the ocean factor, it is easy to ignore the role of the local environment. (2) The climate of the study area is characterized by precipitation and heat during the same period such that there are certain differences in the main ion contents of the two periods. This difference can be ignored when using the mean values for calculations.

Another sensitivity test involves two scenarios of varying silicate end-member compositions. The other reservoirs (precipitation and carbonate) and major elemental composition of the silicate reservoir remained unchanged between the two scenarios, with only variations in the ^87^Sr/^86^Sr ratio of the silicate reservoir. Scenario 5 had ^87^Sr/^86^Sr of 0.720–0.80 while Scenario 6 had ^87^Sr/^86^Sr of 0.708–0.910 to take into account unradiogenic basalt and volcanic rocks. The two scenarios produced similar results (i.e., approximately 1% difference in α_sil, Cation_ and a 3–25% difference in α_sil, Cation_ while α_sil, Cation_ represents the proportional coefficient of chemical weathering derived from silicate rock). When the end-member compositions cover a larger range, we obtained better chemical weathering assessment results. However, using a narrowly defined component for the end-member may produce false results^41^. According to the results of the path model analysis, silicate was a non-negligible structural factor in the spatial distribution of Sr and its isotopes. The difference in the weathering and dissolution characteristics between the two periods may have caused the different spatial distribution of Sr and its isotopes in the Xijiang River Basin. Therefore, we suggest that Scenario 6, with a wider range, is a better choice.

In addition, the sensitivity test had a set of collected precipitation end-member data with higher (X/Na^2+^) ratios, i.e., HCO_3_^−^/Na^+^ = 245.33, Mg^2+^/Na^+^ = 4.73, Ca^2+^/Na^+^ = 31.93, and Cl^−^/Na^+^ = 8.25. We suggest that these data are abnormal and not suitable for analytical calculations. Therefore, blind use of the inversion model has certain problems, but relatively accurate results can be obtained by reducing the uncertainty of the end-member background information. At the same time, by combining Sr and its isotopes with the semi-variance model and path model analysis, we can obtain new techniques to confirm the end-members in the inversion model.

#### Calculation of the carbon flux from the weathering of each end-member

The chemical weathering rate of rocks is closely related to the water temperature, precipitation, rock type, and anthropogenic activity in the basin. Therefore, this rate is regarded as a dynamic process. In this process, the main problem in terms of calculating the CO_2_ flux absorbed by silicate weathering is determining the proportion of sulfuric acid involved in the weathering of silicate and carbonate rocks. The carbonate chemical weathering process is more complicated than that of silicate. In previously reported calculations^40^, the participation of sulfuric acid in carbonate weathering did not fully consider the separate responses of the limestone and dolomite weathering processes to structural and random factors but rather considered both as a whole. In addition, sulfuric acid participates in carbonate chemical weathering to generate equimolar CO_2_ such that this produced CO_2_ returns to the water body to sustain rock weathering. The silicate weathering rate (SWR) was calculated as the total amount of cations from silicate weathering by either carbonic or sulfuric acid:

4 $$\text{SWR}=\upalpha_{\text{sil,Na}}\varPhi{\text{Na}}_{\text{river}}+\upalpha_{\text{sil,K}}\varPhi{\text{K}}_\text{river}+\upalpha_\text{sil,Mg}\varPhi{\text{Mg}}_{\text{river}}+\upalpha_{\text{sil,Ca}}\varPhi{\text{Ca}}_{\text{river}},$$

where *Φ* indicates the yield of different cations (mol/km^2^/year). Equation (4) reflects the exchange reaction of a cation in the silicate with carbonic or sulfuric acid. Here, α_sil, Na_, α_sil, Mg_, and α_sil, Ca_ were derived from the chemical weathering proportionality coefficient of silicate. Silicate weathering in the study area was regarded as a dynamic process that depends on the proportion of sulfate in the river, i.e., the amount of sulfuric acid involved in the chemical weathering of silicate and carbonate. We assumed that, in the extreme case where all sulfates derive from gypsum dissolution that coexists with carbonate, carbonate-induced silicate weathering (CSW) was equal to the flux of CO_2_ consumed by silicate weathering. However, when exposed to anthropogenic activities, sulfuric acid plays a regulatory role in chemical weathering, with no CO_2_ consumption during silicate weathering such that it must be subtracted. The CSW value can be calculated as follows:

5 $${\text{CSW }} = \, \upalpha _{{\text{sil,Na}}}\Phi {\text{Na}}_{{{\text{river}}}} + \upalpha _{{\text{sil,K}}}\Phi {\text{K}}_{{{\text{river}}}} + 2\upalpha _{{\text{sil,Mg}}}\Phi {\text{Mg}}_{{{\text{river}}}} + 2\upalpha _{{\text{sil,Ca}}}\Phi {\text{Ca}}_{{{\text{river}}}} -\updelta *2\Phi {\text{SO}}_{{{\text{4river}}}} ,$$

where δ is the adjustment coefficient of sulfuric acid (ranging from 0 to 1). The proportion of carbonate and silicate weathering that involved sulfuric acid was equal to the contribution ratio of carbonate and silicate to the total dissolved cations.

For carbonate chemical weathering, the carbonate weathering rate (CWR) was calculated as the sum of cations from the weathering of carbonate due to carbonic or sulfuric acid:

6 $${\text{CWR }} = \, \upalpha _{{\text{car,Na}}}\Phi {\text{Na}}_{{{\text{river}}}} + \, \upalpha _{{\text{car,Mg}}}\Phi {\text{Mg}}_{{{\text{river}}}} + \, \upalpha _{{\text{car,Ca}}}\Phi {\text{Ca}}_{{{\text{river}}}} ,$$

where α_car,Na_, α_car,Mg_, and α_car,Ca_ are the output coefficients of carbonate calculated by the inversion model. The contribution ratio between limestone and dolomite to the carbonate cations is represented by β. The rock weathering rate and related CO_2_ flux of limestone and dolomite can be further obtained with the contribution ratio of limestone and dolomite to the total dissolved cations.

The CO_2_ absorption due to carbonic acid weathered carbonate can be calculated as follows:

7 $${\text{CO}}_{{{\text{2car}}}} = \, 0.5 \, \times \, \left( {\left[ {{\text{HCO}}_{3} } \right]_{{{\text{river}}}} {-}{\text{ CSW}}} \right).$$

The corresponding equation for the carbonic acid weathered carbonate (CCW) is as follows:

8 $${\text{CCW }} = {\text{ CO}}_{{{\text{2car}}}} .$$

The limestone weathering rate (LWR) was calculated as the sum of the cations from weathering due to carbonic or sulfuric acid:

9 $${\text{LWR}} = \, \upalpha _{{\text{lim,Na}}} \Phi {\text{Na}}_{{{\text{river}}}} + \, \upalpha _{{\text{lim,Mg}}}\Phi {\text{Mg}}_{{{\text{river}}}} + \, \alpha_{{\text{lim,Ca}}}\Phi {\text{Ca}}_{{{\text{river}}}} .$$

The corresponding dolomite weathering rate (DWR) can be expressed as:

10 $${\text{DWR}} = {\text{ CWR }}{-}{\text{ LWR}}.$$

Furthermore, we can calculate the CO_2_ produced by sulfuric acid weathered carbonate (SCW) with the following expression:

11 $${\text{SCW }} = \, \left( {1 \, {-} \,\updelta } \right) \, * \,\Phi {\text{SO}}_{{{\text{4river}}}} .$$

The corresponding CO_2_ generated by the chemical weathering process of limestone due to sulfur acid (SLW) can be calculated as follows:

12 $${\text{SLW }} = \,\upbeta \, *{\text{ SCW}}.$$

The CO_2_ produced by the chemical weathering of dolomite due to sulfur acid (SDW) can be expressed as:

13 $${\text{SDW }} = \, \left( {1 \, {-} \,\upbeta } \right){\text{SCW}}.$$

In Eq. (13), β is the adjustment coefficient of limestone and dolomite in carbonate. The ratio of limestone and dolomite weathering that involved sulfuric acid is equal to the contribution ratio of limestone and dolomite to the total dissolved cations.

The consumed CO_2_ in the limestone chemical weathering process due to carbonic acid (CLW) can be expressed as:

14 $${\text{CLW }} = \,\upbeta \, *{\text{ CCW}}.$$

The consumed CO_2_ in the dolomite chemical weathering process due to carbonic acid (CDW) can be expressed as:

15 $${\text{CDW }} = {\text{ CCW }} - {\text{ CLW}}.$$

## Results

Table 3 lists the main ionic contents and Sr and its isotopes in the water samples from the mainstream of the Xijiang River and its tributaries measured in June 2014 and January 2015.

**Table 3:** Chemical compositions of the main ions at each sampling point of the mainstream and tributaries of Xijiang River.

River name	Sample no	Sampling date	T (ºC)	pH	K^+^	Na^+^	Ca^2+^	Mg^2+^	Cl^−^	SO_4_^2−^	HCO_3_^−^	SiO_2_ (mg/L)	Sr (mg/L)	TDS (mg/L)	^87^Sr/^86^Sr
					mmol/L										
Hongshui river	XJ01	June 2014	24.47	7.83	0.03	0.12	1.44	0.25	0.09	0.13	2.92	5.82	0.17	266.92	0.709909
Honghsui river	XJ02		24.44	7.83	0.03	0.13	1.51	0.28	0.11	0.25	3.07	5.65	0.17	292.62	0.708452
Hongshui river	XJ03		23.69	7.76	0.03	0.13	1.52	0.29	0.12	0.28	3.07	6.10	0.18	296.82	0.708453
Hongshui river	XJ04		24	7.81	0.04	0.15	1.49	0.31	0.10	0.21	3.01	5.09	0.2	283.94	0.708454
Hongshui river	XJ05		28.63	8.55	0.03	0.12	1.14	0.22	0.08	0.14	2.19	4.30	0.14	209.15	0.708457
Hongshui river	XJ06		29.58	8.78	0.04	0.21	0.91	0.38	0.13	0.37	1.94	2.06	0.24	213.08	0.708448
Hongshui river	XJ07		24.24	7.77	0.05	0.23	1.49	0.45	0.15	0.42	2.88	5.37	0.28	303.93	0.708902
Gui river	XJ08		28.3	8.40	0.03	0.07	0.69	0.14	0.09	0.11	1.44	7.45	0.044	142.29	0.711541
He river	XJ09		25.8	8.44	0.04	0.13	1.09	0.26	0.19	0.16	2.38	6.33	0.062	227.84	0.711493
He riber	XJ10		27.5	8.36	0.03	0.10	0.55	0.14	0.10	0.11	1.19	9.82	0.04	124.96	0.711459
Gui river	XJ11		26.8	8.31	0.02	0.06	0.64	0.13	0.08	0.10	1.38	7.62	0.04	135.64	0.710887
Xijiang	XJ12		27.1	8.23	0.03	0.09	0.74	0.15	0.10	0.12	1.44	8.52	0.06	148.13	0.712861
Xijiang	XJ13		27.1	7.91	0.04	0.10	0.86	0.17	0.13	0.15	1.69	8.18	0.083	172.59	0.708783
Qianjiang	XJ14		25.5	8.28	0.03	0.10	0.92	0.18	0.12	0.17	1.76	8.07	0.10	180.26	0.709618
Yujiang	XJ15		28.7	8.23	0.06	0.17	1.30	0.22	0.24	0.17	2.63	8.80	0.083	258.03	0.710329
Yujiang	XJ16		28.1	8.20	0.05	0.11	1.41	0.21	0.18	0.16	2.73	8.13	0.087	262.13	0.710605
Zuo river	XJ17		27.8	8.25	0.03	0.07	1.50	0.20	0.12	0.14	2.95	7.96	0.076	273.02	0.711363
Zuo river	XJ18		28.8	8.34	0.04	0.08	0.21	0.17	0.14	0.13	2.38	8.35	0.078	186.68	0.711047
Zuo river	XJ19		29.0	8.53	0.03	0.08	1.20	0.18	0.09	0.11	2.57	9.70	0.08	235.65	0.710306
Heishui river	XJ20		28.0	8.56	0.02	0.09	1.54	0.22	0.10	0.25	3.01	6.16	0.075	287.00	0.710334
Hongshui river	XJ01	January 2015	17.41	7.95	0.04	0.16	1.43	0.34	0.09	0.33	1.34	3.92	0.247	188.1	0.708426
Hongshui river	XJ02		17.69	8.04	0.04	0.16	1.46	0.35	0.09	0.32	2.86	4.02	0.229	280.87	0.708449
Hongshui river	XJ03		17.73	7.74	0.04	0.16	1.42	0.36	0.09	0.32	2.86	2.43	0.213	279.02	0.708454
Hongshui river	XJ04		18.16	8.05	0.04	0.16	1.41	0.36	0.09	0.32	2.89	4.12	0.23	280.64	0.708451
Hongshui river	XJ05		17.74	7.91	0.04	0.16	1.42	0.36	0.09	0.33	2.92	1.05	0.248	283.7	0.708457
Honghsui river	XJ06		17.35	8.07	0.04	0.17	1.41	0.37	0.10	0.34	2.83	3.57	0.267	279.71	0.708456
Honghsui river	XJ07		17.28	7.83	0.04	0.18	1.43	0.39	0.10	0.35	2.90	3.67	0.261	286.93	0.70844
Gui river	XJ08		14.76	7.97	0.04	0.15	0.82	0.17	0.13	0.12	1.75	2.58	0.0534	164.7	0.712445
He river	XJ09		12.94	7.75	0.05	0.12	1.13	0.31	0.15	0.15	2.45	1.74	0.0534	226.81	0.710637
He river	XJ10		12.74	7.88	0.05	0.17	0.73	0.19	0.19	0.12	1.63	3.23	0.0514	157.57	0.712348
Gui river	XJ11		13.58	7.88	0.05	0.15	0.78	0.17	0.12	0.12	1.69	3.32	0.0568	159.18	0.712763
Xijiang river	XJ12		13.54	7.81	0.05	0.18	1.04	0.23	0.14	0.21	2.22	4.91	0.121	212.91	0.709798
Xijiang river	XJ13		15.92	8.00	0.05	0.19	1.20	0.28	0.15	0.25	2.51	4.71	0.156	243.49	0.708934
Qianjiang	XJ14		17.27	7.90	0.04	0.17	1.25	0.30	0.11	0.03	2.86	4.22	0.21	243.78	0.708632
Yujiang	XJ15		17.56	7.82	0.07	0.30	1.30	0.20	0.24	0.15	2.74	5.55	0.0685	256.38	0.710603
Yujiang	XJ16		18.85	7.77	0.05	0.16	1.25	0.21	0.13	0.15	2.68	4.86	0.0817	243.1	0.710054
Zuojiang	XJ17		17.53	7.68	0.05	0.18	1.12	0.18	0.14	0.11	2.33	6.44	0.0609	212.86	0.711155
Zuojiang	XJ18		17.89	7.81	0.04	0.14	1.00	0.18	0.10	0.03	2.33	6.00	0.0603	197.46	0.71157
Zuojiang	XJ19		17.20	7.86	0.04	0.16	0.95	0.17	0.08	0.08	2.22	7.78	0.0696	192.62	0.710364
Heishui river	XJ20		17.12	7.88	0.03	0.12	1.47	0.24	0.08	0.03	3.09	3.87	0.0594	262.62	0.710248

Based on Table 3, the pH of the mainstream and tributaries in the high-water period ranged from 7.76 to 8.78, with an average of 8.21. In the low-water period, the pH ranged from 7.68 to 8.07, with an average value of 7.88. The river water was alkaline in both periods due to the chemical weathering of carbonate and dolomite rocks. There was no significant change in the total dissolved solids (TDS), i.e., ranging from 124.96 to 266.92 mg/L in the high-water period (mean: 225.03 mg/L) and 157.57–286.93 mg/L in the low-water period (mean: 232.62 mg/L). At the same time, the river water type was Ca-HCO_3_ in both carbonate and non-carbonate areas. Here, HCO_3_^−^ was the main anion, ranging from 1.19 to 3.07 mmol/L in the high-water period and1.34–3.09 mmol/L in the low-water period. The secondary anion in the river water was SO_4_^2−^ (0.10–0.42 and 0.03–0.35 mmol/L, respectively) while the corresponding range of Cl^−^ was 0.08–0.24 mmol/L in both periods. Together, SO_4_^2−^ and HCO_3_^−^ accounted for more than 80% of the anions in most river water samples. Meanwhile, Ca and Mg were the major cations, accounting for more than 80% of the cations in nearly all water samples. The only exception was sample XJ18 from the Zuojiang River, where the main components were Na^+^ and K^+^. At the same time, the Ca^2+^/Mg^2+^ molar ratio at XJ18 was only 1.24 in the high-water period (far lower than the other points) and 5.56 in the low-water period. The reasons for this phenomenon are complicated. First, XJ18 is situated in the middle reaches of the Zuojiang River, with abundant thick-layered limestones and a small amount of dolomite. This lithology can explain why Ca^2+^ and Mg^2+^ became the main cations in this area. Furthermore, the Xijiang River Basin is a region characterized by numerous agricultural activities from April–September, which may increase the Na^+^ and K^+^ levels during the high-water period. However, finding a definitive explanation for the changes in the Ca^2+^/Mg^2+^ molar ratio is difficult. Nevertheless, Sr and its isotopic compositions were relatively stable at XJ18, rather than reflecting the drastic change in the cation compositions during the high- and low-water periods. Perhaps the combination of anthropogenic activity and stratigraphic lithology leads to certain masked information between Sr and its isotopes. In other words, the weathering end-members of Sr isotopes derive from relatively similar sources, which can more effectively reflect the origin of river solutes. In certain cases, however, complex lithology (i.e., multiple lithologic interbeds) or anthropogenic activity can interfere with the Sr isotopic compositions, rendering them less accurate.

## Discussion

### Spatial characteristics of Sr and its isotopes

The content of Sr in different sources was significantly different while its chemical properties were stable. At the same time, as Sr isotopes are not affected by material fractionation, the ^87^Sr and ^86^Sr sample compositions can reflect characteristics of the river water environment and aquifer^50^, which were mainly derived from rock weathering and input from anthropogenic activity. The Sr isotope ratio is a parameter related to climate change when applied to the chemical weathering process. The water body mixing process and interactions among water, rock, and particles are typically inferred by the Sr isotope ratio and ion ratio in water^51^. However, these results usually only indicate the range in the samples, such as the type of weathering and magnitude of the influencing factors, rather than providing feedback on the spatial distribution characteristics of Sr and its isotopes. As a result, spatial information and variation rules are often neglected. The dual factors of complex lithologic distributions and anthropogenic activities influence the actual situation, providing more natural and social attributes to Sr and its isotopes (social refers to the information that can be fed back through Sr and its isotopes under the influence of anthropogenic activities, e.g., increases or decreases in the value of Sr and its isotope composition may be due to contamination from anthropogenic activities). Therefore, the above description of Sr is not rigorous. First, in areas with complex lithology, carbonate and silicate weathering occurs simultaneously, which may obscure the trends of Sr and its isotopic ratio in the river (i.e., the obtained Sr values cannot provide sufficient feedback on the two weathering situations). Second, rock weathering is a complex dynamic process that changes with the environment such that samples only reflect the situation within a certain period of time and given environment. Third, anthropogenic activities have become a non-negligible factor affecting the environment, including Sr and its isotope ratio. If only the numerical values of Sr and its isotopes are used to characterize rock weathering in a certain area, the obtained conclusions may not describe the entire situation. To solve this problem, the gstat package in the R language was used for the semi-variance analysis of Sr and its isotopes in the Xijiang River water to explore the underlying spatial characteristics. Table 4 lists the parameters of the semi-variance theoretical model. For comparison, the spatial autocorrelation range (variable range) in the table was converted to km. The substrate effect refers to the nugget/sill ratio (a higher ratio indicates a larger local variability).

**Table 4:** Semi-variance function of Sr and its isotopes and the model’s fitting parameters.

Date	Characteristic	Theoretical model	Nugget	Sill	Range (km)	Nugget/Sill (%)
June 2014	Sr	Gauss	0.000267	0.00897	222	2.98
June 2014	^87^Sr/^86^Sr	Bessel	3.21e−07	8.18e−06	371	3.92
January 2015	Sr	Hole	0.00209	0.00575	63	36.3
January 2015	^87^Sr/^86^Sr	Hole	1.27e−06	7.75e−07	66	62.1

The semi-variance theoretical models of Sr and its isotopes in the high-water period were Gaussian and Bessel models, respectively, while that in the low-water period were void models. In general, when the nugget/sill ratio was less than 25%, structural factors were dominant. The random factors became dominant when this ratio was greater than 75%. Both factor sets had substantial effects when the ratio was between 25 and 75%. The nugget/sill ratio for Sr increased from 2.98 to 36.3% from the high- to low-water periods (the spatial autocorrelation range decreased from 222 to 63 km), with an increase in the nugget/sill ratio of ^87^Sr/^86^Sr from 3.92 to 62.1% (the spatial autocorrelation range decreased from 371 to 66 km). These results indicate that both structural and random factors in the two periods effected the spatial variability of Sr and its isotopes in the study area. In the high-water period, structural factors mainly affected the spatial variability of Sr and its isotopes, with strong spatial autocorrelation. The effect of structural factors noticeably weakened in the low-water period. Random factors, such as anthropogenic activities, caused a decrease in the spatial autocorrelation and range of variation controlled by structural factors. In summary, both structural and random factors affected Sr and its isotopes^52^.

### Factors affecting the spatial structure of Sr and its isotopes

#### Types of river weathering

In nearly all large global rivers, chemical weathering is a combination of silicate and limestone weathering, even in a monolithologic catchment with a pure geological background. However, when runoff flows through certain karst areas, there is a significant difference, i.e., the appearance of dolomite weathering. According to Brass^52^, Table 5 lists the Sr concentration and isotope ratio of the limestone, dolomite, and silicate end-members. In addition, the molar ratios of Mg/Ca, Na/Ca, Mg/Sr, Ca/Sr, Na/Sr, and HCO_3_/(HCO_3_ + SO_4_) in the tributaries of the Xijiang River were 0.1–0.8, 0.04–0.39, 122.06–500.34, 230.79–2166.77, and 0.8–1.0, respectively. The source of water can be identified by the corresponding relationships among the molar ratios.

**Table 5:** Molar ratios of the river end-members.

	Mg^2+^/Ca^2+^	Na^+^/Ca^2+^	Mg^2+^/Sr	Ca^2+^/Sr	Na^+^/Sr	^87^Sr/^86^Sr	HCO_3_^**−**^/(HCO^**−**−^ + SO_4_^2**−**^)
Limestone	~ 0.1	~ 0.02	40–50	~ 350	> 10	~ 0.7075	~ 0.7
Dolomite	~ 1.1	~ 0.02	~ 2000	~ 2000	> 100	~ 0.711	~ 0.9
Silicate	0.4–0.8	~ 5	~ 200	~ 200	> 700	> 0.715	0.8–0.9

**Figure 2:**
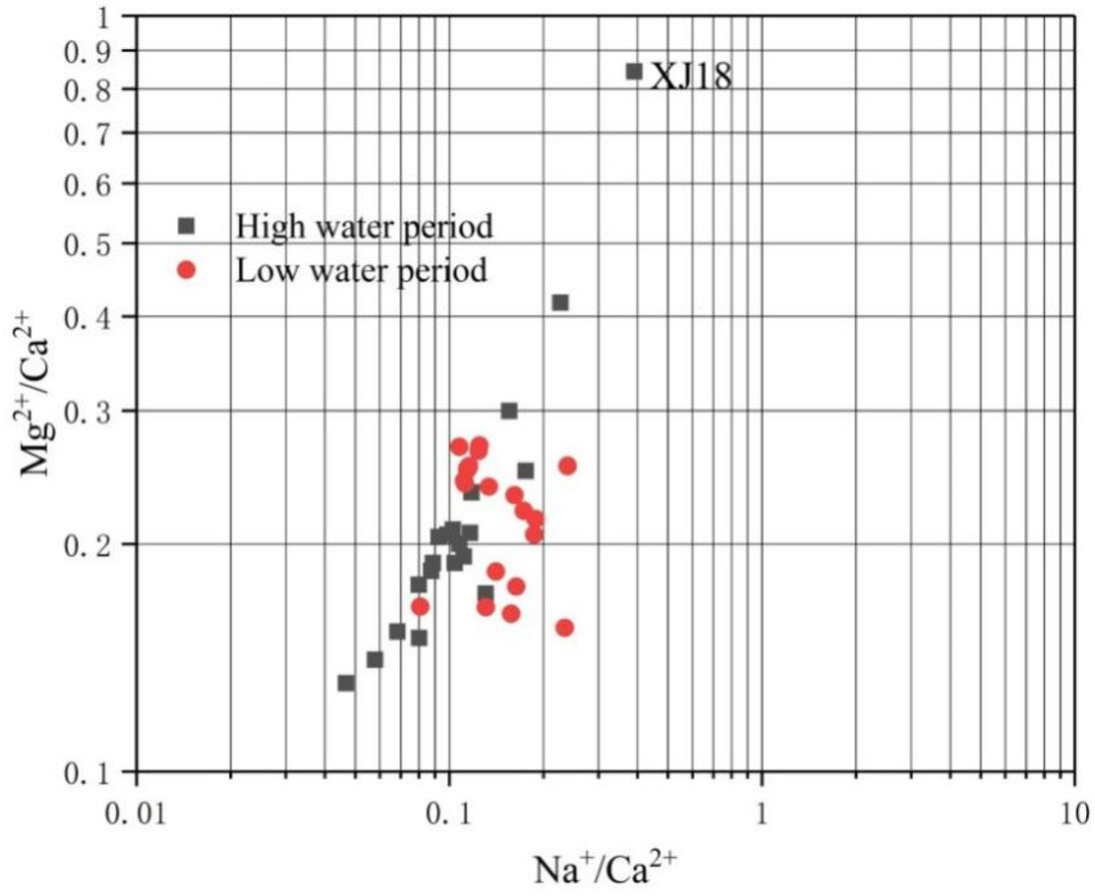
The relationship between Mg^2+^/Ca^2+^ and Na^+^/Ca^2+^ ratios in river water during the high (grey squares) and low (red circles) water periods on a log–log scale.

Figure 2 shows the relationship between Mg^2+^/Ca^2+^ and Na^+^/Ca^2+^ in the Xijiang River. The temperature of the mainstream and tributaries ranged from 10.42 to 29.0 °C, and the Mg^2+^/Ca^2+^ ratio was predominantly less than 0.8. This may be due to the equilibrium among river water, calcite, and dolomite based on spontaneous reactions at room temperature. Thus, calcite and dolomite are in equilibrium at room temperature when Mg^2+^/Ca^2+^ is 0.8 (Palmer and Edmond, 1989). When point XJ18 was in the high-water period, the Mg^2+^/Ca^2+^ ratio was higher such that the characteristics of dissolution and balance between calcite and dolomite were more evident. Therefore, elemental ratios in river water indicate that the rock weathering substances roughly originated from three sources: limestone, dolomite, and silicate rocks.

#### Differences in water temperature

The changes in the water temperature between the high- and low-water periods and water temperature can affect rock weathering through the pH and abundance of planktonic microorganisms in water^53^ such that there are corresponding changes in the values of Sr and its isotopes. The Pearson correlation coefficients (R^2^) between water temperature and Sr and its isotopes in the high-water period were − 0.469 and 0.360, respectively, while in the low-water period they were 0.452 and − 0.567, respectively. Here, the R^2^ values had opposite signs between the two periods, indicating that variations in the water temperature can cause a corresponding change in the value of Sr and its isotopes.

**Figure 3:**
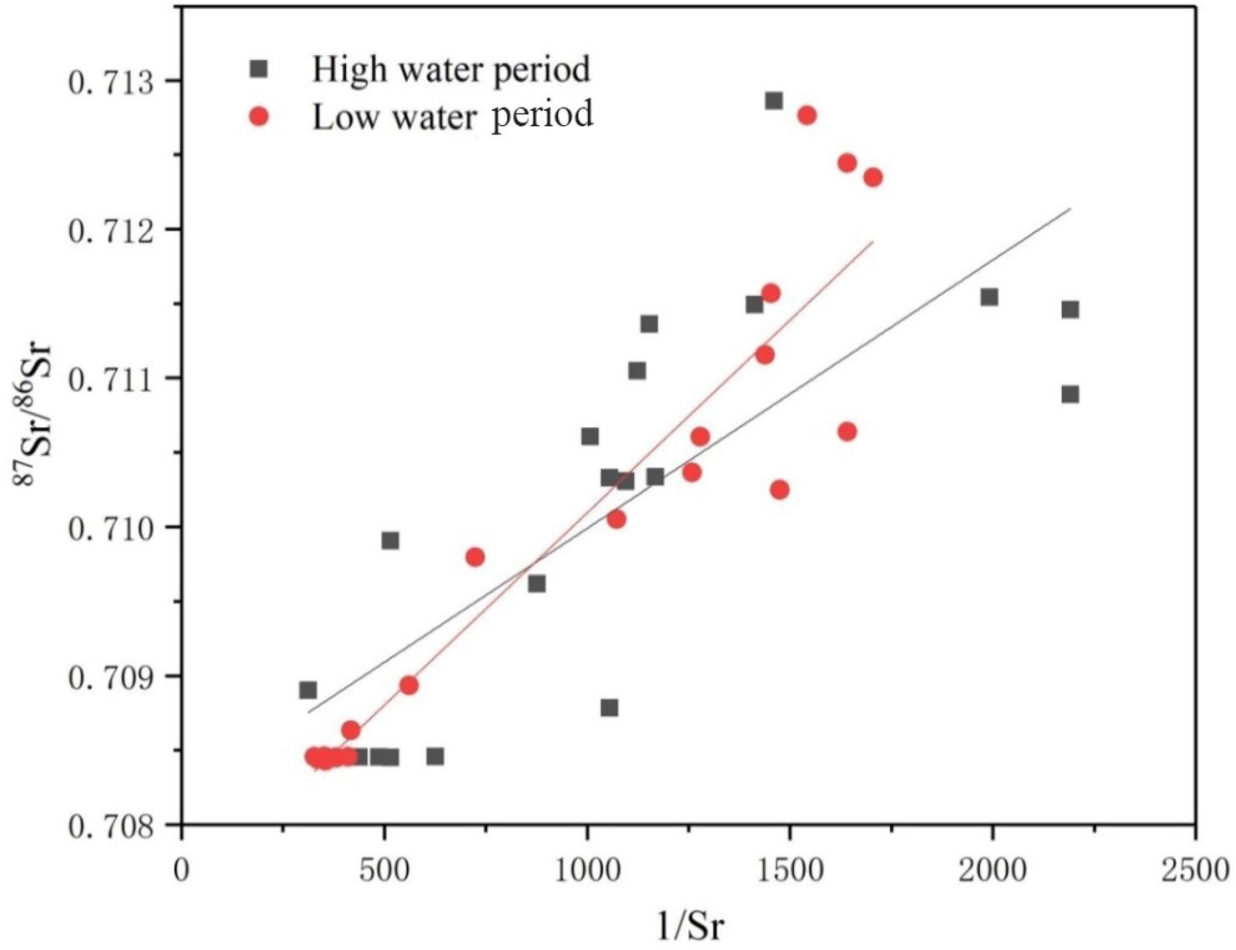
The relationship between ^87^Sr/^86^Sr and 1/Sr during the high (grey squares) and low (red circles) water periods.

Previous studies have shown that the ^87^Sr/^86^Sr ratio in river water tends to fall between 0.7045 and 0.943^54^, with relatively low compositions in carbonate areas (0.706–0.709) and elevated Sr concentrations. In contrast, rivers that drain silicate have relatively radiogenic ^87^Sr/^86^Sr ratios (generally higher than 0.710) while their Sr concentrations are relatively low. The ^87^Sr/^86^Sr ratios in the study area varied between 0.7084 and 0.7129, with a mean value of 0.710, which is lower than the average global river composition (0.7119). The dissolved Sr ranged from 0.46 to 3.2 μmol/L, with an average of 1.5 μmol/L, which is significantly higher than the average global river concentration (0.89 μmol/L). These findings are consistent with the presence of widespread carbonates in the study area. In addition, a positive correlation between ^87^Sr/^86^Sr and 1/Sr in the river water may indicate that strontium from both carbonate and silicate sources caused the changes in the ^87^Sr/^86^Sr for soluble strontium in the river water (Fig. 3)^55^. The R^2^ values in the high- and low-water periods were 0.602 and 0.880, respectively, indicating that carbonate and silicate weathering affected both the mainstream and tributaries. In other words, limestone, dolomite, and silicate weathering mainly control the ^87^Sr/^86^Sr ratios of the river water, which is consistent with previous studies.

#### Random factors

The element sources in the river water are atmospheric deposition, rock weathering, and human activities. The pollution of human activities are mainly imported into the water body through two ways: atmospheric input and human discharge (industrial sewage, pesticide, and fertilizer residues). The water was characterized by anthropogenic products. It is rich in four elements such as K, S, Cl, and N and K, S, and Cl, which are also the products of soil/rock weathering. The Xijiang River Basin has a wide area and a large east–west span. It is the main agricultural and mining area in Guangxi. The agricultural soil area is approximately 40,000 km^2^. According to the type of farmland land use, it is mainly dry land and paddy field. Dry land has a rich variety of crops. There are various types of pesticides or fertilizers. The main crops in paddy fields are rice, and the application of pesticides and fertilizers is relatively simple. The Hechi area in the upper reaches of the river basin is an important non-ferrous metal production base in southwestern China. The Nandan area is rich in arsenic. Its reserves are 27% and 19% of national and global reserves, respectively. Wuxuan County in the middle and lower reaches has an important lead–zinc deposit; however, the mineral resources in the Xijiang Basin are mainly in the mining stage, and many mines are already in the integration and stop-mining stage. Thus, pollution is limited, and the impact on the environment is negligible. There are no other industries in the study area besides mining, and therefore, it can be considered that the industries in the study area are not developed^56^. N exists in water bodies in the form of NO_3_^−^, mainly derived from nitrogen fertilizers used in agricultural activities, whereas SO_4_^2−^ in surface water mainly comes from industrial activities and atmospheric input. Because the industry in the study area is underdeveloped, the impact is small, and therefore, it can be considered that SO_4_^2−^ in rainwater is mainly derived from atmospheric input.

**Figure 4:**
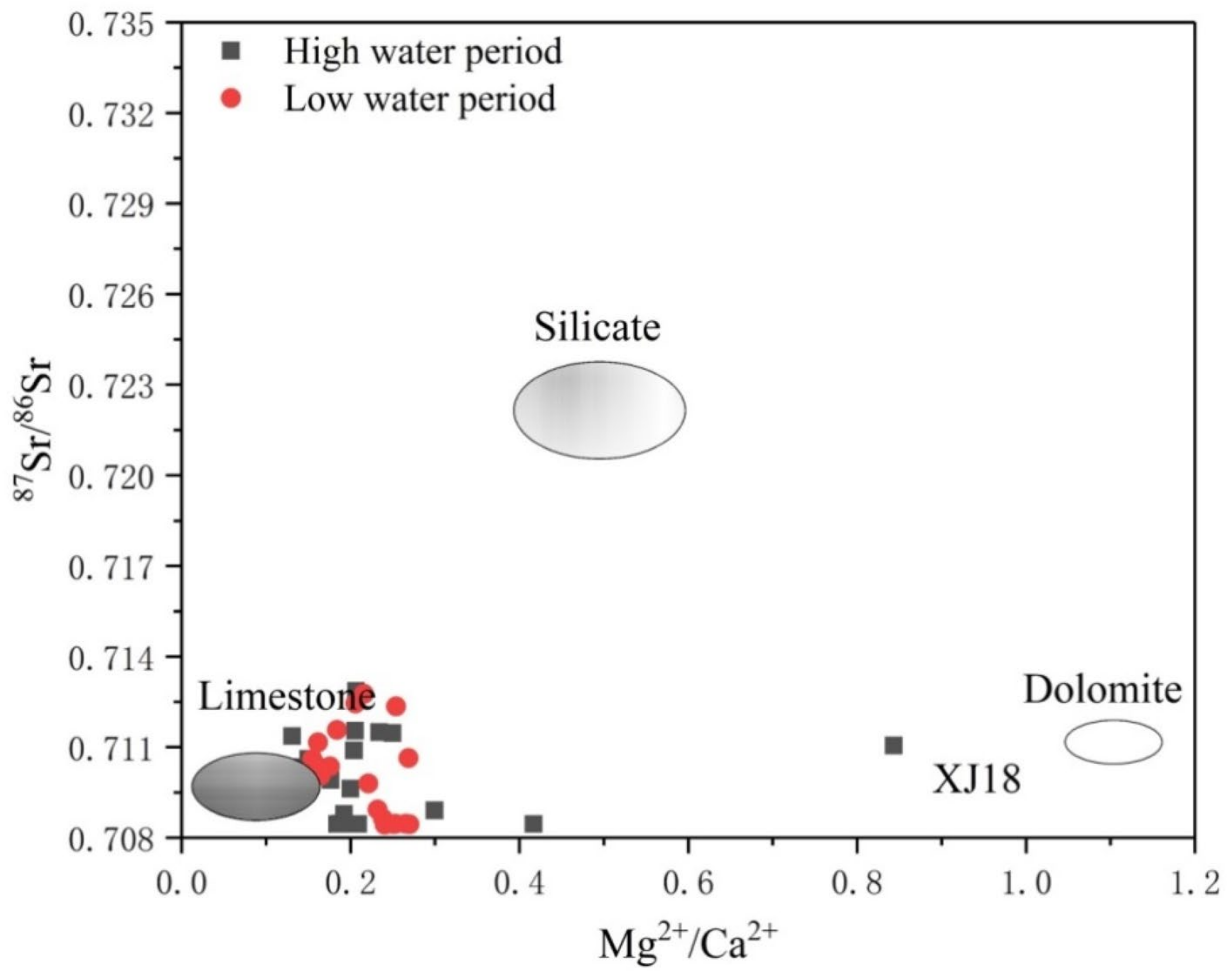
The relationship between the ^87^Sr/^86^Sr and Mg^2+^/Ca^2+^ ratios in river water of the mainstream and tributaries. The oval fields represent the different end-members. If a sample point falls within one ellipse, then it is controlled by the corresponding end-member. If it falls between two or three ellipses, then it is controlled by both or all end-members.

Figure 4 shows that, although the chemical weathering of limestone, silicate, and dolomite effected most samples, the main control was limestone weathering. However, these results cannot be attributed to the influence that human activity has on Sr and its isotope ratio. For this reason, we used stoichiometric analyses to further identify the anthropogenic activities.

**Figure 5:**
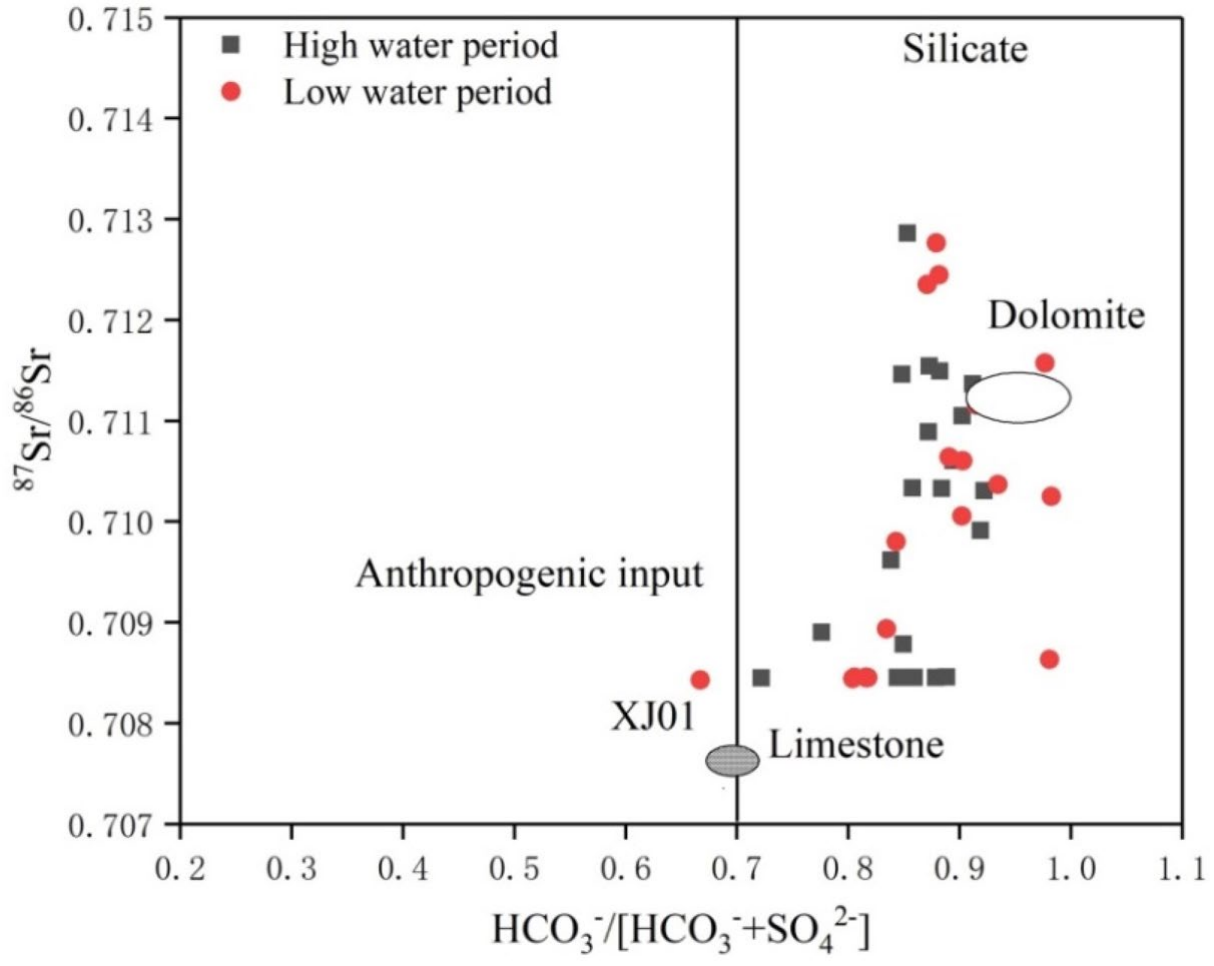
The relationship between ^87^Sr/^86^Sr and HCO_3_^−^/[HCO_3_^−^ + SO_4_^2−^] in river water during the high (grey squares) and low (red circles) water periods.

**Figure 6:**
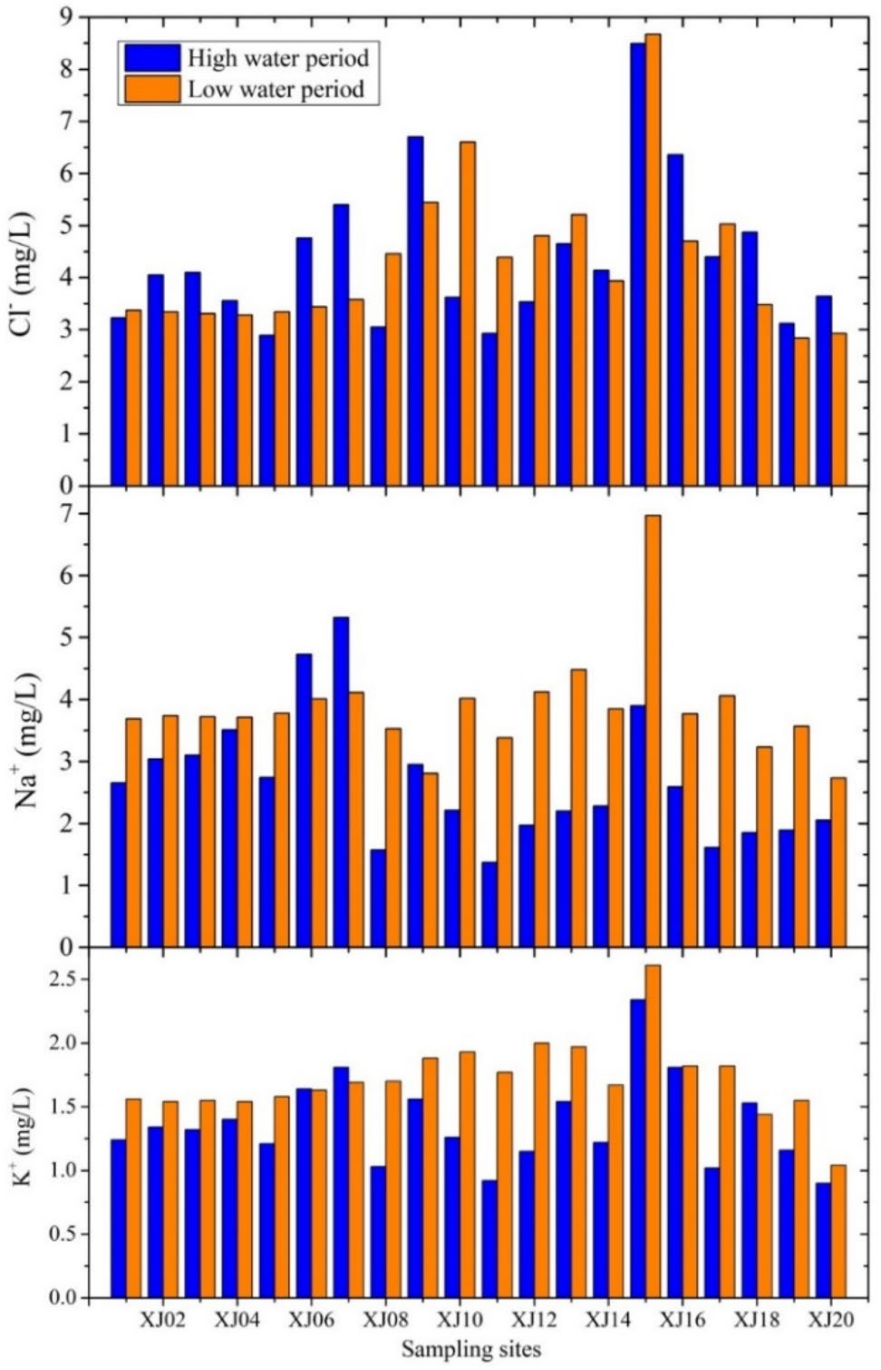
Variations in the K^+^, Na^+^, and Cl^−^ concentrations of the Xijiang River during the high (in blue) and low (in red) water periods.

As shown in Fig. 5, the relationship between the ^87^Sr/^86^Sr and HCO_3_^−^/[HCO_3_^−^ + SO_4_^2−^] ratios in the Xijiang mainstream and tributaries yields information on the weathering of dolomite, limestone, and silicate. The HCO_3_^−^/[HCO_3_^−^ + SO_4_^2−^] equivalence ratio was greater than 0.7 for most river water samples. Anthropogenic activities significantly affected certain river samples (XJ01). To evaluate the influence that anthropogenic activities have on Sr and its isotopes, we used the [Ca^2+^  + Mg^2+^]/HCO_3_^−^ equivalence ratio. For most rivers, this ratio is greater than 1 while the mean ratios in the study area during the high- and low-water periods were 0.98 and 1.22, respectively. In the high-water period, this ratio was less than 1, indicating that ionic equilibrium required additional cations, such as K^+^ and Na^+^. Apart from natural weathering, K^+^ may also derive from anthropogenic activities. In the low-water period, the [Ca^2+^  + Mg^2+^]/HCO_3_^−^ equivalent ratio was greater than 1, indicating that the equilibrium involved other anions, i.e., most likely NO_3_^−^ produced by anthropogenic activities. In summary, there were significant differences in water stoichiometry within the study area. We can infer that anthropogenic activities had a certain degree of influence on the chemical composition of water in the Xijiang River. Further analyses found that the concentrations of K^+^, Na^+^, and Cl^−^ in the study area had similar changes in time and space (Fig. 6), indicating that there was a certain correlation between these ions in water. Among these ions, Cl^−^ is a conservative element as it does not easily fractionate. Although mainly controlled by sea salt sedimentation, anthropogenic activities also had certain effects. For K^+^, the study area has developed agriculture with a high rate of potassium fertilizer application. At the same time, there are numerous reservoirs in the study area such that the regulation of anthropogenic activities effects Na^+^, i.e., sodium ions mainly derive silicate weathering, which is typically a slower process than carbonate weathering. The retention time of a water body is longer because reservoirs render silicate weathering to be a more congruent process. This suggests that anthropogenic activities effected the Xijiang mainstream and its tributaries.

**Figure 7:**
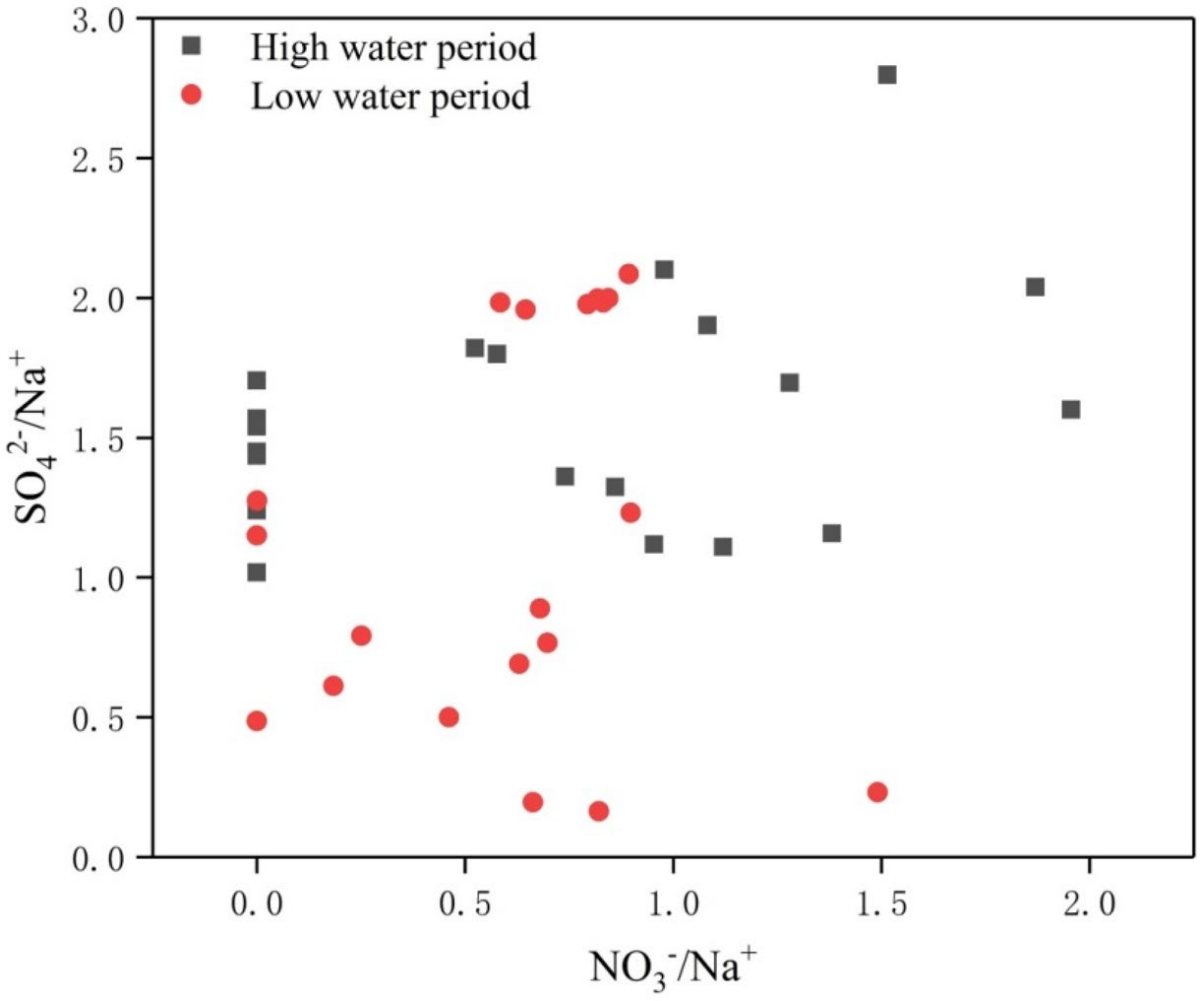
The relationship between SO_4_^2−^/Na^+^ and NO_3_^−^/Na^+^ during high (grey squares) and low (red circles) water periods in the tributaries of the Xijiang River.

Whether or not the SO_4_^2−^ and NO_3_^−^ derive from the same source can be determined based on the relationship between SO_4_^2−^/Na^+^ and NO_3_^−^/Na^+^ (Fig. 7). There is no significant correlation between these two ratios, indicating that the SO_4_^2−^ and NO_3_^−^ have different sources. First, SO_4_^2−^ in river water mainly derives from rock weathering, gypsum dissolution, sulfide oxidation, atmospheric precipitation, and anthropogenic activities^1^. As NO_3_^−^ mainly derives from anthropogenic input, we can infer that SO_4_^2−^ mainly originates from geological effects. Second, the study area is essentially free of evaporites and coal-bearing strata. Therefore, river SO_4_^2−^ mainly derives from the paragenesis of gypsum in the carbonate strata, i.e., gypsum is slightly soluble in water. In addition, although the amount of sulfuric acid produced by anthropogenic SO_2_ was 3–5 times that of natural pyrite, this can only increase the ion concentration in the river water by approximately 13%, indicating that there was no significant effect on the CO_2_ consumed by karstification^57^. In other words, sulfate produced by anthropogenic activities has little effect on the chemical weathering signature in water bodies. Therefore, we suggest that the anthropogenic source of SO_4_^2−^ is minor while that from geological sources (mainly carbonate and gypsum symbiosis) is the major contributor. In other words, the structural factors are dominant, whereas the random factors are not evident. The NO_3_^−^ concentrations were low in the study area, even zero for several samples. Therefore, we only considered the effect that acid formed by CO_2_ and SO_2_ in water has on chemical weathering, which can be described by the following equation:

16 $$3{\text{Ca}}_{{(1{-}{\text{x}})}} {\text{Mg}}_{{\text{x}}} \left( {{\text{CO}}_{3} } \right) \, + {\text{ HCO}}_{3}^{-} + {\text{ H}}_{2} {\text{SO}}_{4} = \, 3{\text{x}}\,{\text{Ca}}^{2 + } + \, 3\left( {1 \, {-}{\text{ x}}} \right){\text{ Mg}}^{2 + } + \, 4{\text{HCO}}_{{3}}^{-} + {\text{SO}}_{4}^{{2{-}}}$$

**Figure 8:**
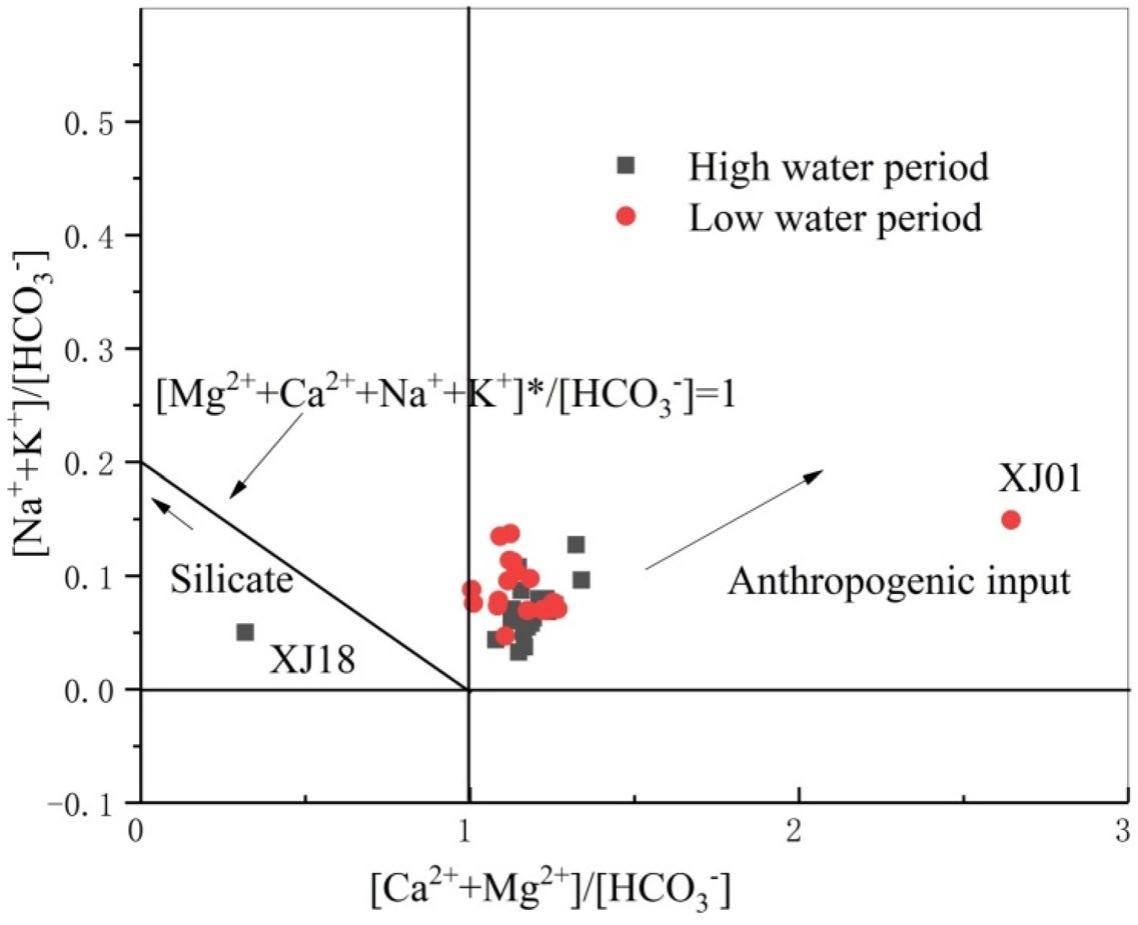
The elemental ratios of silicate and carbonate weathering for river water from the high (in grey squares) and low (in red circles) water periods.

As sulfuric acid participates in chemical weathering, SO_4_^2−^ balanced a portion of the [Ca^2+^  + Mg^2+^] in the water, assuming that SO_2_ derived from atmospheric input acted only on the equilibrium of [Ca^2+^  + Mg^2+^]. The quantity of [Ca^2+^  + Mg^2+^]^*^([Ca^2+^  + Mg^2+^]^*^ = [Ca^2+^  + Mg^2+^] – [SO_4_^2−^]) was then derived from carbonate or silicate weathering. Therefore, the [Ca^2+^  + Mg^2+^]^*^/[HCO_3_^−^] ratio (value of less than 1) can represent the relative contents of Ca^2+^ and Mg^2+^ in carbonate and silicate weathering. Similarly, [Na^+^  + K^+^]^*^([Na^+^  + K^+^]^*^ = [Na^+^  + K^+^] − [Cl^−^]) in river water represents the Na^+^ and K^+^ from carbonate and silicate weathering. Based on this, the relationship between the changes in carbonate weathering (i.e., [Ca^2+^  + Mg^2+^]^*^/[HCO_3_^−^] and [Na^+^  + K^+^]^*^/[HCO_3_^−^]) was plotted (Fig. 8) to reflect the relative contributions of carbonate and silicate weathering to the river water solutes. Most samples fall into the first quadrant (i.e., both [Ca^2+^  + Mg^2+^]^*^ and [Na^+^  + K^+^]^*^ exceed the HCO_3_^−^ content), which indicates that there were other cations in addition to those produced by carbonate and silicate chemical weathering. These excess cations likely derive from atmospheric input. At the same time, these cations may exist in equilibrium with Cl^−^ or NO_3_^−^. When the proportion of excess ions is small, as shown in Fig. 8, the influence that SO_2_ has on weathering can be determined based on Eq. (16): 3 mol of (Ca_x_Mg_1–x_)CO_3_ requires 1 mol of H_2_SO_4_ and H_2_CO_3_ for a reaction, with a SO_4_^2−^/HCO_3_^−^ equivalence ratio of 0.5. The averages of the SO_4_^2−^/HCO_3_^−^ equivalence ratios in the tributaries of the Xijiang River during the high and low-water periods were 0.161 and 0.162, respectively, which are much smaller than the theoretical value (0.5).

### Path model analysis

According to the previous discussion, the spatial distribution of Sr and its isotopes can reflect the difference between the structural and random factors in the high and low-water periods. This difference and the influence that various factors have on the spatial distribution of Sr and its isotopes were not exactly the same, thus requiring further analysis. Considering that classic hydrogeochemical and common statistical methods have certain limitations in their abilities to discriminate the factors that influence Sr isotopes, the path model was used in this study to additionally analyze the influencing factors for Sr and construct a network system for Sr and its isotopes (Table 6). This allows a more intuitive identification of the direct and indirect influences that each of the causal factors have on Sr and its isotopes.

**Table 6:** Comparison of classic discriminant methods and the path model in hydrogeochemistry for studying chemical weathering.

Model or method	Application scope
SPSS	The correlation between ion and hydrochemical indicators can be used to identify the source of substances, the relationship between two variables, and the direction of correlation. However, the degree of correlation between the two variables cannot be accurately obtained
Classic hydrogeochemical ratio	Judging whether a water body is within a certain weathering range or degree based on the ratio between one or more ions or isotopes
Path model	Characterization of the correlation among various variables performed in a more visual and intuitive manner using the main ions and saturation index of the water

**Figure 9:**
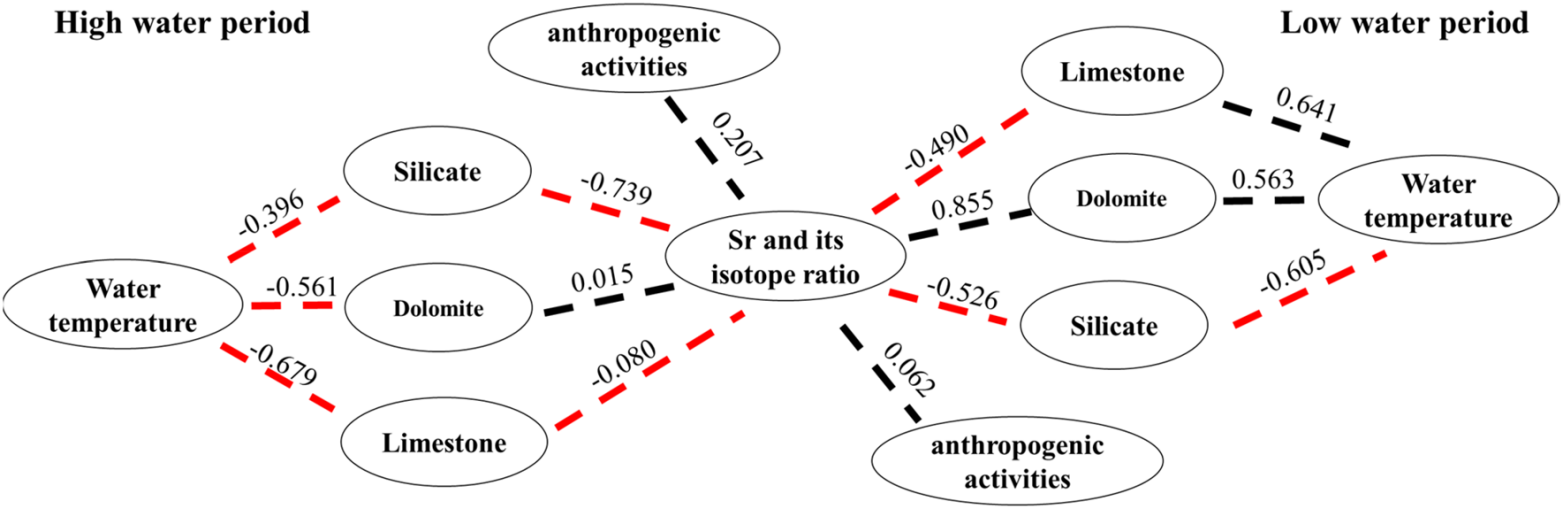
The path model of Sr and its isotopes for various factors during periods of high (left) and low water (right).

The saturation index was calculated using the measured water chemistry data and the PHREEQC2.5.1 water chemistry program (Table 7). We then constructed the PLS-PM model (Fig. 9). In the discriminant method, if the value between two factors is negative, the causal relationship is then opposite, and vice versa. A larger absolute value indicates a stronger causal relationship while the magnitude of the values indicates the degree of intensity.

**Table 7:** The main ion concentrations and saturation indices in the study area for the (a) high- and (b) low-water period.

Date	Sample nos.	T (ºC)	K^+^ (mg/L)	Na^+^ (mg/L)	Ca^2+^ (mg/L)	Mg^2+^ (mg/L)	Cl^−^ (mg/L)	SO_4_^2−^ (mg/L)	HCO_3_^−^ (mg/L)	SiO_2_ (mg/L)	NO_3_^−^ (mg/L)	SIA	SIC	SID	SIG	Sr (mg/L)	^87^Sr/^86^Sr
**(a) High-water period**																	
2014.6.20	XJ01	24.47	1.24	2.65	57.65	6.11	3.23	12.37	177.85	5.82	6.81781	− 3.16	− 0.28	− 1.19	− 2.94	0.17	0.709909
2014.6.24	XJ02	24.44	1.34	3.04	60.32	6.67	4.05	24.13	187.42	5.65	8.87124	− 2.82	− 0.25	− 1.11	− 2.6	0.17	0.708452
2014.6.24	XJ03	23.69	1.32	3.1	60.72	6.88	4.10	27.18	187.42	6.10	8.181	− 2.81	− 0.33	− 1.27	− 2.59	0.18	0.708453
2014.6.25	XJ04	24	1.40	3.51	59.4	7.45	3.56	19.94	183.59	5.09	7.00903	− 2.96	− 0.29	− 1.14	− 2.73	0.2	0.708454
2014.6.25	XJ05	28.63	1.21	2.74	45.72	5.18	2.89	13.24	133.87	4.30	10.20259	− 3.19	0.28	0	− 2.99	0.14	0.708457
2014.6.26	XJ06	29.58	1.64	4.73	36.29	9.08	4.76	35.95	118.57	2.06	6.67934	− 2.86	0.35	0.5	− 2.66	0.24	0.708448
2014.6.26	XJ07	24.24	1.81	5.32	59.44	10.69	5.40	39.96	175.94	5.37	8.27563	− 2.67	− 0.35	− 1.11	− 2.45	0.28	0.708902
2014.6.29	XJ08	28.3	1.03	1.57	27.71	3.42	3.05	10.09	87.97	7.45	< 0.05	− 3.46	− 0.24	− 1	− 3.26	0.044	0.711541
2014.6.20	XJ09	25.8	1.56	2.95	43.58	6.12	6.70	15.26	145.34	6.33	< 0.05	− 3.16	0.15	− 0.2	− 2.94	0.062	0.711493
2014.6.20	XJ10	27.5	1.26	2.21	21.87	3.28	3.62	10.23	72.67	9.82	6.67326	− 3.54	− 0.46	− 1.38	− 3.33	0.04	0.711459
2014.6.20	XJ11	26.8	0.92	1.37	25.79	3.16	2.93	9.7	84.15	7.62	4.72778	− 3.5	− 0.38	− 1.32	− 3.28	0.04	0.710887
2014.6.21	XJ12	27.1	1.15	1.97	29.42	3.65	3.53	11.92	87.97	8.52	< 0.05	− 3.38	− 0.4	− 1.33	− 3.16	0.06	0.712861
2014.6.23	XJ13	27.1	1.54	2.2	34.38	3.97	4.65	14.4	103.27	8.18	< 0.05	− 3.24	− 0.59	− 1.75	− 3.03	0.083	0.708783
2014.6.23	XJ14	25.5	1.22	2.28	36.82	4.42	4.14	16.22	107.09	8.07	< 0.05	− 3.18	− 0.2	− 0.97	− 2.96	0.10	0.709618
2014.6.24	XJ15	28.7	2.34	3.9	51.94	5.36	8.49	16.56	160.64	8.80	< 0.05	− 3.06	0.09	− 0.41	− 2.85	0.083	0.710329
2014.6.25	XJ16	28.1	1.81	2.59	56.27	5.07	6.36	15.52	166.38	8.13	< 0.05	− 3.06	0.03	− 0.6	− 2.85	0.087	0.710605
2014.6.25	XJ17	27.8	1.02	1.61	59.86	4.7	4.40	13.7	179.77	7.96	8.11698	− 3.09	0.2	− 0.31	− 2.88	0.076	0.711363
2014.6.25	XJ18	28.8	1.53	1.85	8.22	4.16	4.87	12.36	145.34	8.35	9.75178	− 3.85	− 0.58	− 1.06	− 3.64	0.078	0.711047
2014.6.26	XJ19	29.0	1.16	1.89	48.1	4.42	3.12	10.44	156.82	9.70	4.38424	− 3.27	0.35	0.06	− 3.07	0.08	0.710306
2014.6.26	XJ20	28.0	0.90	2.05	61.54	5.19	3.64	23.93	183.59	6.16	8.36956	− 2.85	0.52	0.35	− 2.64	0.075	0.710334
**(b) Low-water period**																	
2015.1.16	XJ01	17.41	1.56	3.69	57.22	8.26	3.37	32.12	81.88	3.92	8.88527	− 2.77	− 0.61	− 1.81	− 2.53	0.247	0.708426
2015.1.20	XJ02	17.69	1.54	3.74	58.34	8.5	3.34	30.97	174.44	4.02	5.89378	− 2.79	− 0.18	− 0.94	− 2.54	0.229	0.708449
2015.1.20	XJ03	17.73	1.55	3.72	56.67	8.53	3.31	30.8	174.44	2.43	8.33843	− 2.8	− 0.49	− 1.56	− 2.55	0.213	0.708454
2015.1.20	XJ04	18.16	1.54	3.71	56.41	8.54	3.28	30.94	176.22	4.12	8.18553	− 2.8	− 0.17	− 0.9	− 2.55	0.23	0.708451
2015.1.21	XJ05	17.74	1.58	3.78	56.82	8.64	3.34	31.54	178	1.05	8.6	− 2.79	− 0.31	− 1.19	− 2.54	0.248	0.708457
2015.1.22	XJ06	17.35	1.63	4.01	56.24	8.96	3.44	32.77	172.66	3.57	6.97882	− 2.78	− 0.17	− 0.9	− 2.53	0.267	0.708456
2015.1.22	XJ07	17.28	1.69	4.11	57.25	9.27	3.58	33.92	177.11	3.67	8.79813	− 2.76	− 0.4	− 1.35	− 2.51	0.261	0.70844
2015.1.15	XJ08	14.76	1.7	3.53	32.86	4.06	4.46	11.29	106.8	2.58	6.64	− 3.39	− 0.72	− 2.14	− 3.14	0.0534	0.712445
2015.1.17	XJ09	12.94	1.88	2.81	45.39	7.32	5.44	14.45	149.52	1.74	6.80219	− 3.19	− 0.7	− 2.03	− 2.94	0.0534	0.710637
2015.1.17	XJ10	12.74	1.93	4.02	29.28	4.46	6.6	11.6	99.68	3.23	6.82792	− 3.42	− 0.92	− 2.48	− 3.17	0.0514	0.712348
2015.1.16	XJ11	13.58	1.77	3.38	31.19	4.04	4.39	11.17	103.24	3.32	2.29047	− 3.41	− 0.86	− 2.42	− 3.16	0.0568	0.712763
2015.1.17	XJ12	13.54	2	4.12	41.43	5.5	4.8	19.78	135.28	4.91	< 0.05	− 3.08	− 0.71	− 2.11	− 2.83	0.121	0.709798
2015.1.20	XJ13	15.92	1.97	4.48	48.19	6.71	5.21	23.85	153.08	4.71	0.01	− 2.95	− 0.37	− 1.38	− 2.71	0.156	0.708934
2015.1.21	XJ14	17.27	1.67	3.85	50.08	7.16	3.94	2.64	174.44	4.22	8.52463	− 3.89	− 0.38	− 1.35	− 3.65	0.21	0.708632
2015.1.20	XJ15	17.56	2.61	6.97	51.84	4.82	8.67	14.15	167.32	5.55	< 0.05	− 3.15	− 0.46	− 1.7	− 2.91	0.0685	0.710603
2015.1.22	XJ16	18.85	1.82	3.77	50.1	4.96	4.7	13.99	163.76	4.86	6.91131	− 3.16	− 0.51	− 1.76	− 2.92	0.0817	0.710054
2015.1.23	XJ17	17.53	1.82	4.06	44.83	4.35	5.03	10.37	142.4	6.44	2.0147	− 3.32	− 0.73	− 2.22	− 3.08	0.0609	0.711155
2015.1.23	XJ18	17.89	1.44	3.23	39.87	4.4	3.48	2.64	142.4	6.00	5.77358	− 3.95	− 0.63	− 1.96	− 3.71	0.0603	0.71157
2015.1.24	XJ19	17.20	1.55	3.57	37.93	4	2.84	7.45	135.28	7.78	4.44	− 3.52	− 0.63	− 2	− 3.28	0.0696	0.710364
2015.1.23	XJ20	17.12	1.04	2.73	58.77	5.83	2.93	2.64	188.68	3.87	10.9703	− 3.84	− 0.3	− 1.37	− 3.59	0.0594	0.710248

The structural and random factors in the high- and low-water periods have significant influences on the potential variables of Sr and its isotopes. The causal relationship between water temperature and limestone and dolomite was − 0.679 and − 0.561 in the high-water period, and 0.641 and 0.563 in the low-water period, respectively. Therefore, lower water temperatures in the low-water period increase the solubility of calcite and dolomite, which promotes the weathering and dissolution of limestone and dolomite^58^. The causal relationship between water temperature and silicate rocks was − 0.396 and − 0.605, respectively, in the high- and low-water periods. This relationship was relatively larger in the high-water period while the intensity was weaker. This indicates that, during the high-water period, the causal relationship was greater than low-water periods but the intensity of former was weaker than the latter.

Both values were negative, indicating that a higher water temperature in the study area during the high-water period promoted the chemical weathering of silicates^53^. Moreover, the dissolution rate of carbonate due to weathering was higher than that of silicate. On the other hand, the extensive presence of carbonate in the study area inhibited the weathering and dissolution of silicate, resulting in a negative causal relationship between water temperature and silicate weathering.

Based on the path model, the causal relationships for Sr and its isotopes among limestone, dolomite, and silicate during the high-water period were − 0.080, 0.015, and − 0.739, and − 0.490, 0.855, and − 0.526 during the low-water period, respectively. These relationships show that, although the warm and humid climate in the high-water period can accelerate plant degradation, it promotes the chemical weathering of silicates and increases the intensity of chemical weathering. In other words, organic acids formed by plant degradation may accelerate the weathering of silicate rocks. This can also be the case for carbonate but, for the Xijiang River, the water temperature is a structural factor while the role of microorganisms is not necessarily a structural factor. Plant degradation can also contribute to the weathering of carbonate rocks but before structural factors, it is weaker than the water temperature to reduce the dissolution of calcite and dolomite rock. We, therefore, do not consider this situation. However, the causality relationship between silicate and Sr and its isotopes was stronger during the high-water period, indicating that the solubility of calcite and dolomite decreased due to the relatively high water temperature^58^. Therefore, silicate has a relatively stronger influence on Sr and its isotopes during the high-water period.

Although anthropogenic activities also promote the weathering and dissolution of rocks, the corresponding causal relationship with Sr and its isotopes in the high-water period was weaker than that in the low-water period, suggesting that anthropogenic activities have a much weaker influence than water temperature on Sr and its isotopes. The structural factors mainly controlled weathering during the high-water period^59^ such that chemical weathering concealed the effects of anthropogenic activities. For anthropogenic activities, the causal relationship with Sr and its isotopes in the high and low-water periods was 0.207 and 0.062, respectively, which is consistent with the characteristics of agriculturally developed areas in the basin. More pesticides and fertilizers are used in the summer as compared with the winter, resulting in a stronger causal relationship between anthropogenic activities and Sr and its isotopes in the summer. The calculated causal relationships between the structural factors (such as water temperature) and Sr and its isotopes in the high- and low-water periods were − 0.466 and 0.325, respectively. For the study area, the causal relationship between anthropogenic activities in the high-water period was opposite to and weaker than the control by the structural factors. This also indicates that chemical weathering was stronger than anthropogenic activities, with dominant structural factors. However, in the low-water period, the causal relationship for anthropogenic activities had the same direction as that for the structural factors but was much weaker. We can infer that random factors affect the weathering of rocks in the basin together with the structural factors, indicating noticeably enhanced dolomite weathering. In general, the sources of Sr and its isotopes were different during the high- and low-water periods. The weathering and dissolution of silicate dominated the structural factors in the high-water period. In the low-water period, both structural and random factors, i.e., mainly the weathering and dissolution of dolomite, affected Sr and its isotopes.

### Model calculation and verification

**Figure 10:**
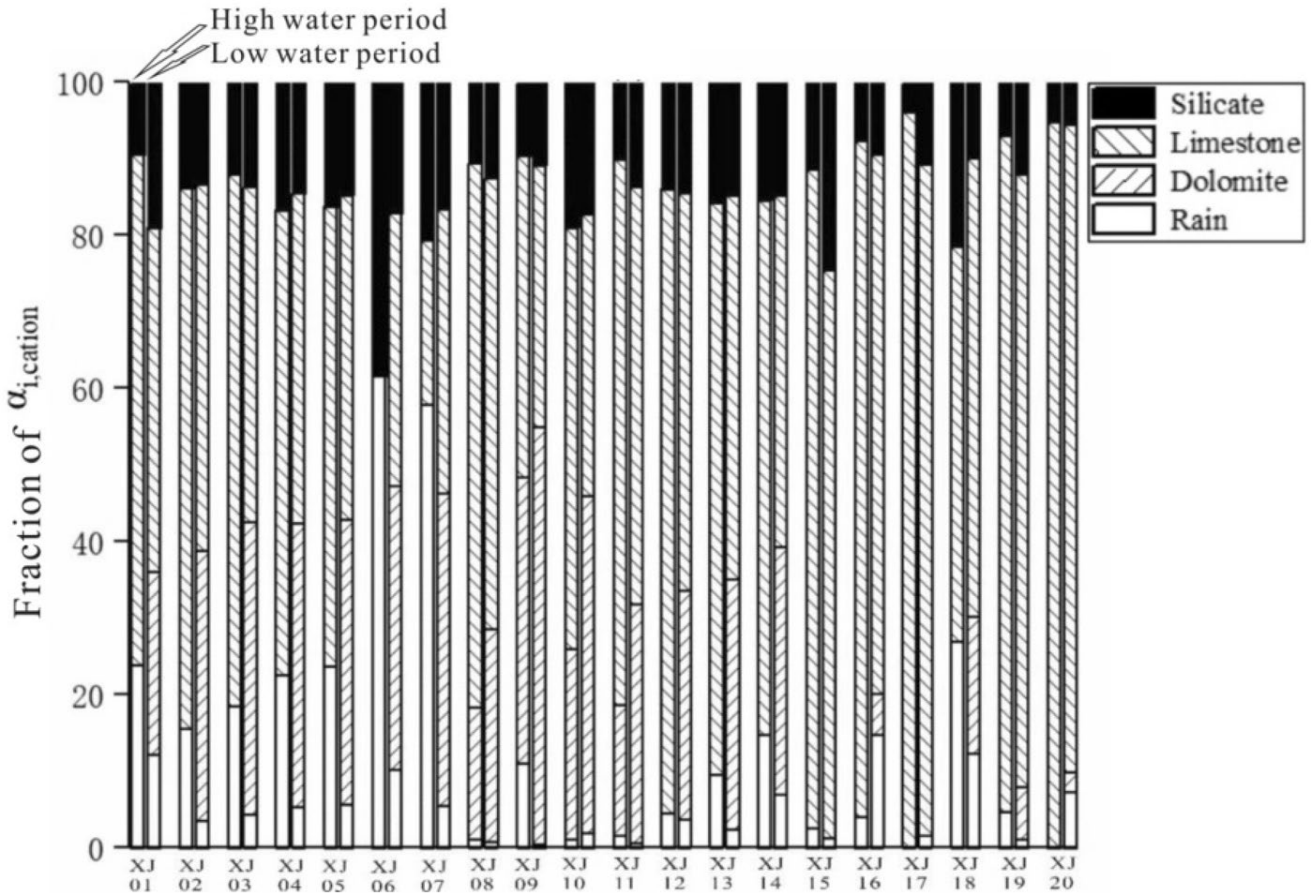
The fraction of total dissolved cations (α_i,Catin =_ α_i,Ca_ + α_i,Na_ + α_i,Mg_ + α_,K_) from rainfall, dolomite, limestone, and silicate. Two bars for the high (left) and low-water period (right) represent each sampling point, as indicated for sample XJ01.

The value of each parameter in the inversion model for the Xijiang River Basin was obtained by iteration (Table 8), whose results are listed in Table 9 and shown in Fig. 10.

**Table 8:** End-member parameters of the model.

End member	Ca/Na	Mg/Na	HCO_3_/Na	Cl/Na	1000 * Sr/Na	^87^Sr/^86^Sr
**Rain**						
High water period	3.83	1.08	23.14	1.41	16.51	0.709
Low-water period	1.66	0.30	13.58	0.61	9.69	0.709
**Carbonate**						
High water period	70.00	12.18	137.95	0.001	50.00	0.7088
Low-water period	69.32	17.56	132.34	0.001	50.00	0.7087
**Silicate**						
High water period	0.56	0.62	1.17	0.001	3.06	0.7910
Low-water period	0.56	0.39	1.00	0.001	10.06	0.7804

**Table 9:** Calculated dissolved fluxes for the Xijiang River.

Sample nos.	Area (km^2^)	Runoff (mm/year)	Discharge (km^3^/year)	Source of cation (mol%)				TDS yield (10^6^ mol/km^2^/year)	SWR		Net CO_2_ yield
									10^3^ mol/km^2^/year		ΦCation_sil_
				Rain	Dolomite	Limestone	Silicate		Inverse	2φSi	10^3^ mol/km^2^/year
**(A) High water period**											
Hongshui river											
XJ01	4364.18	20,016	87.35	23.8	0.0	66.8	9.4	103.79	3103.67	3883.16	3998.46
XJ02	4088	10,800	44.15	15.5	0.0	70.7	13.8	60.78	3131.40	2034.00	4049.69
XJ03	112,500	566	63.7	18.5	0.0	69.5	12.0	3.22	135.84	115.14	162.06
XJ04	112,200	5588	627	22.5	0.0	61	16	30.73	1965.99	948.14	2671.43
XJ05	106,580	5207	555	23.8	0.0	59.9	16.3	21.66	1341.32	746.39	1820.19
XJ06	98,500.00	5249	517	61.5	0.0	0.0	38.5	21.67	3164.52	360.41	1444.77
XJ07	3196.00	4174	13.34	57.9	0.0	21.5	20.6	24.56	1816.40	747.12	1264.21
Gui river											
XJ08	3273.00	4095	13.4	1.1	0.0	88.3	10.6	11.03	378.41	1016.91	460.47
XJ11	2989.00	169,866.00	507.73	1.6	1.7	86.7	10.0	46.57	15,006.69	43,145.97	15,986.00
Xijiang river											
XJ12	10,606.00	26,903.00	285.34	4.5	0.0	81.7	13.8	987.29	4090.89	7640.57	4728.85
XJ13	22,112.00	1982.00	43.84	9.6	0.0	74.7	15.7	556.54	387.59	540.54	459.21
Yujiang											
XJ15	8633.30	2203.00	19.02	2.6	0.0	86.1	11.3	132.81	382.95	646.11	360.75
XJ16	7265.60	2917.00	21.19	4.0	0.0	88.5	7.5	9.88	288.76	790.45	264.31
Zuo river											
XJ17	111,855.00	5179.00	67.80	0.0	0.0	96.1	3.9	114.96	149.59	1517.52	85.68
XJ18	2682.30	1376.00	3.69	26.9	0.0	51.6	21.5	7.24	55.06	382.87	0.00
XJ19	4281.00	1738.00	7.44	4.7	0.0	88.3	7.0	12.9	159.31	562.11	176.54
He river											
XJ09	2680.00	18,357.00	49.20	11.1	3.7	75.6	9.6	24.91	62,138.25	114,925.81	69,957.40
XJ10	6380.00	351,097.00	2240.00	1.0	2.5	77.5	19.0	3.28	15,006.69	43,145.97	15,986.00
Qianjiang											
XJ14	1966.55	22,354.00	43.96	14.8	0.0	69.8	15.4	5.94	4506.28	6013.35	5470.14
Heishui river											
XJ20	1213.81	3429.00	4.16	0	0	95	5	18.29	242.89	704.19	259.87
**(B) Low-water period**											
Hongshui river											
XJ01	4364.18	4003.26	17.47	12.1	3.4	65.5	19.0	15.84	1538.78	523.09	1918.08
XJ02	4088	2160.00	8.83	3.5	5.4	77.8	13.3	11.78	609.27	289.44	691.70
XJ03	112,500	113.25	12.74	4.3	6.0	76.1	13.6	0.61	31.59	9.17	35.76
XJ04	112,200	1117.65	125.40	5.3	5.7	74.4	14.6	6.11	330.56	153.49	383.25
XJ05	106,580	1041.47	111.00	5.6	5.7	74.0	14.7	5.71	313.12	36.45	361.64
XJ06	98,500.00	1049.75	103.40	10.2	5.9	66.8	17.1	5.71	359.52	124.92	424.22
XJ07	3196.00	834.78	2.67	5.5	6.49	71.5	16.5	4.67	296.29	102.12	345.89
Gui river											
XJ08	3273.00	818.99	2.68	0.8	3.8	82.8	12.6	2.64	48.28	70.43	25.19
XJ11	2989.00	33,973.21	101.55	0.7	4.5	81.3	13.5	107.53	3848.06	3759.70	3088.23
Xijiang river											
XJ12	10,606.00	5380.69	57.07	3.7	4.3	77.6	14.4	22.64	1033.47	880.64	942.72
XJ13	22,112.00	396.48	8.77	2.4	4.9	78.1	14.6	1.87	102.83	62.25	103.07
Yujiang											
XJ15	440.53	3.80	57.07	1.2	0.0	74.3	24.5	2.24	230.74	81.50	280.67
XJ16	583.36	4.24	8.77	14.7	0.5	74.3	24.5	2.75	30.68	94.50	13.51
Zuo river											
XJ17	1143.86	13.56	8.79	1.6	0.0	87.7	10.7	4.86	53.39	245.55	26.57
XJ18	275.12	0.74	3.8	12.3	2.1	75.7	9.9	1.10	13.82	55.02	0.00
XJ19	347.70	1.49	4.24	1.2	0.7	86.3	11.8	1.35	41.55	90.17	39.82
He river											
XJ09	3671.36	9.84	13.56	0.5	8.9	79.8	16.13	5.02	653.87	212.94	700.15
XJ10	70,219.44	448.00	0.74	1.9	7.1	73.8	228.00	0.86	12,343.94	7560.29	11,244.20
Qianjiang											
XJ14	4470.89	8.79	1.49	7.0	4.9	73.4	14.7	21.59	1134.31	628.91	1587.98
Heishui river											
XJ20	685.90	0.83	0.83	7.2	0.3	87.0	5.5	3.64	18.32	88.48	16.08

In the main stream and tributaries during the high-water period, the percentages of cations that derive from precipitation, dolomite, limestone, and silicate were 15.3% (0.0–61.5%), 0.4% (0.0–3.7%), 70.5% (0.0–96.1%), and 13.8% (3.9–38.5%), respectively. In the low-water period, these values were 5.1% (0.5–14.7%), 4.0% (0.0–8.9%), 77.0% (65.5–87.7%), and 13.9% (5.5–24.5%), respectively. The results show that the proportion of ions from the carbonate weathering process was the largest, with limestone as the main source, which is mostly due to the distribution of different types of bedrock in the basin. Most samples have a TDS flux on the order of 10^6^ mol/km^2^/year, i.e., 96.01 (3.22–871.74) $\times$ 10^6^ mol/km^2^/year in the high-water period and 23.32 (1.10–228.00) $\times$ 10^6^ mol/km^2^/year in the low-water period.

Table 8 indicates that the difference between the basic elemental ratio and Sr isotope ratio of the carbonate end-member during the high- and low-water periods was small while there was a large difference in the Sr isotope value for the silicate end-member. There were several differences in the source of cationic materials in the river during the high- and low-water periods (Table 9).

The results show that limestone was the main material source of water cations in the Xijiang River Basin. The material input from silicate was relatively stable while the cation inputs from the end-members of precipitation and dolomite showed significant differences between the high- and low-water periods.

The study area is near the South China Sea, characterized by high temperature and rain in the summer and limited precipitation and arid conditions in the winter. The unique climate and geological conditions provide different natural sources of chemical substances in the basin. During the high-water period, rainfall was abundant. By analyzing the spatial structure of Sr and its isotopes, we have found that the main influencing factors are structural while random factors caused by anthropogenic activities should have little effect. Therefore, the ocean affected the solutes in rainwater (i.e., the marine impact was attributed to structural factors) such that the source of river solute during the high-water period had an elevated rainfall end-member input. In the low-water period, the dry climate reduced the material input from rainfall compared with the high-water period. The spatial characteristics of Sr and its isotopes reflect the influence of structural and random factors. We suggest that the ocean weakened the contribution from rainfall solutes during the low-water period, whereas this was strengthened by anthropogenic activities. Therefore, variations existed in the input to river water from each end-member. The main solute source in the river was carbonate, contributing up to 70.9% of the cations in the high-water period and 81.0% in the low-water period. Among the different carbonate sources, the weathering and dissolution of limestone was the main contributor in both periods, accounting for 70.5 and 77.0% of cations in the river during the high and low-water periods, respectively. This is due to a number of related factors that affect chemical weathering. First, a large portion of the study area is covered by carbonate rocks (i.e., 44% of the basin area, of which limestone accounts for approximately 40%). The dissolution rate of carbonate due to weathering was much higher than that of silicate^59^, making carbonate the main solute source in the Xijiang River. Second, the weathering dissolution rate of dolomite was greater than that of limestone at the same temperature but both rates decreased with increasing temperature, shrinking the gap between them. The area of limestone in the basin was much larger than that of dolomite. During the high-water period, anthropogenic activities promoted the weathering and dissolution of both limestone and dolomite. However, the effect of anthropogenic activities was weaker than that of the structural factors. According to the path model analysis, under the combined effects of structural and random factors, the causal relationship between limestone and dolomite and Sr and its isotopes was − 0.08 and 0.015, respectively. The causal relationship of limestone was stronger than that of dolomite, which shows that the weathering and dissolution of limestone had a stronger effect. Finally, the decreased water temperature during the low-water period promoted the weathering dissolution of limestone and dolomite^58^, resulting in a higher contribution ratio of carbonate to the other river cations than during the high-water period. Compared with the high-water period, the weathering dissolution rate of dolomite was higher than that of limestone in the low-water period, during which period the impact of anthropogenic activities was relatively higher. According to the results of the path model analysis (“Results” section), the causal relationship for both the anthropogenic activities and control direction of the structural factors was the same, which promotes both the weathering and dissolution of limestone and dolomite. As a result, the weathering solubility of dolomite had a noticeable enhancement compared with that during the high-water period. However, as the dolomite area is much smaller than the limestone area, its material contribution rate was relatively lower.

The silicate weathering rate (SWR) was selected to verify the accuracy of the model. The model calculation yielded SWR values of 5215.37 (55.06–62,138.25) $\times$ 10^3^ and 1151.62 (13.82–12,343.94)$\times$ 10^3^ mol/km^2^/year for the high- and low-water periods, respectively, i.e., fourfold higher in the high-water period. In addition, the uncertainty associated with SWR propagation was approximately 40%, where approximately 30% of the uncertainty was due to α_sil_, _Cation_ and river runoff.

In the absence of quantified cation concentrations from a silicate source, we calculated the SWR by simply assuming that the only source of Si was the weathering of silicates, ignoring potential links between Si and biogeochemical cycles in river water. This assumption leads to a general relationship between the SWR and Si flux in the river (SWR = 2ΦSi). The calculated SWR for the Xijiang River Basin during the high-water period was 9529.70 (115.14–114,925.81)$\times$ 10^3^ mol/km^2^/year, which was higher than that of the inversion model. The value for the low-water period was 753.45 (9.17–7560.29)$\times$ 10^3^ mol/km^2^/year, less than the results of the inversion model. The t-test, based on the SWR = 2ΦSi method at the 5% significance level, yielded an annual CO_2_ flux from weathered silicate after carbonation that was higher than that in the inversion model. Although the linear correlation was significant (R^2^ = 0.987), the difference between the two methods was not significant. Therefore, the paired t-test was performed again for samples in the high- and low-water periods, resulting in a small difference between the two methods at the different time periods. We suggest that the weathering of silicate was a relatively stable process mainly controlled by runoff. The method based on SWR = 2ΦSi, whose original use was for rivers flowing through igneous or metamorphic rocks, yielded the high CO_2_ flux consumed by the CSW (i.e., the chemical weathering process between silicate and carbonic acid). Here, the Si/HCO_3_ ratio was 0.3–0.5. However, the sample used for this calculation had a lower Si/HCO_3_ ratio (0.012–0.138). In addition, we did not consider the action intensity between the structural and random factors in the high- and low-water periods. Therefore, the SWR = 2ΦSi method was relatively inaccurate (Table 9), which is the reason for differences when using these two methods.

Therefore, we must perform further comprehensive calculations of the CO_2_ flux absorbed by silicate weathering. Silicate weathering in the study area should be regarded as a dynamic process that depends on the proportion of sulfate in the rivers, i.e., how much sulfuric acid participates in the chemical weathering of silicates and carbonates. In the extreme case, where all sulfate derives from the dissolution of gypsum coexisting with carbonate, the CSW was equal to the flux of CO_2_ consumed by silicate weathering. However, when exposed to anthropogenic activity, sulfuric acid controls the chemical weathering such that no CO_2_ is consumed during silicate weathering. Thus, we must reduce the SCW flux. Assuming that all negative feedbacks of sulfuric acid on inorganic carbon act on carbonate weathering, the value of δ is 0 (Fn.4), yielding CSW values in the high- and low-water periods of 7228.56 (55.51–84,313.52)$\times$ 10^3^ and 1408.93 (15.27–14,230.66) $\times$ 10^3^ mol/km^2^/year, respectively. This is equivalent to the case where the above sulfate completely originates from gypsum dissolution. Similarly, if the negative feedback of sulfuric acid on inorganic carbon acts on silicate weathering (δ = 1), the CSW value of several tributaries would be negative, which is inconsistent with the actual situation. Therefore, the contribution ratios of carbonate and silicate to the total dissolved cation can be replaced by the weathering ratio of sulfate carbonate to silicate. Final calculations yielded CSW values during the high-water period of 5758.70 (0.00–69,957.40) × 10^3^ mol/km^2^/year and 1112.11(13.56–11,244.20) × 10^3^ mol/km^2^/year during the low-water period. The ΦCO_2_ during the high-water period was approximately fivefold that in the low-water period. The assumed average value of δ differed by approximately 15% from ΦCO_2_, which indicates that carbonation weathering mainly affects the Xijiang River Basin while sulfuric acid plays a secondary role.

In the path model analysis of “Results” section, the causal relationship between silicate and Sr and its isotopes during the high-water period was stronger than that in the low-water period. Based on the inversion model, we assumed that the same input from silicate produces the river cations in both the high- and low-water periods. Our verification calculations show that the silicate ^87^Sr/^86^Sr signature from silicate weathering during the high-water period was relatively higher, i.e., identical to the path model analysis. Therefore, for the Xijiang River Basin, when the same material delivered silicate to the river, a radiogenic ^87^Sr/^86^Sr ratio during the high-water period resulted in a significant causal relationship with Sr and its isotopes. This indicates that silicates had a greater impact during the low-water period because the carbonate was more sensitive to runoff than silicate. At the same time, due to the influence of anthropogenic activities, the ^87^Sr/^86^Sr ratio decreased even when the amount of material input remained the same.

### End-member weathering rates and fluxes

Table 10 lists the fluxes of each end-member in the study area. The total CO_2_ flux consumed by petrochemical weathering was 150.69 × 10^9^ mol/year, i.e., 100.37 and 44.10 × 10^9^ mol/year for the high- and low-water periods (66 and 34% of the total flux), respectively. The gap in the flux is similar to the results for the Li River^42^. The flux ratio was similar to the flow ratio (i.e., the mean monthly flow during the high-water period was 9987.47 km^3^, which was 3.51-fold that during the low-water period, i.e., 2813.62 km^3^). Therefore, the water cycle was the main controlling factor on the carbon sink effect of the Xijiang River Basin. The CO_2_ flux of SCW was 15.40 × 10^9^ mol/year, characterized by an evident reduction in the sink.

**Table 10:** CO_2_ consumption for the Xijiang mainstream and main tributaries.

Acids	Rocks	Weathering type	Weathering intensity: high water period 10^3^ mol/km^2^/year	Weathering intensity: low-water period 10^3^ mol/km^2^/year	Flux: high water period 10^9^ molCO_2_	Flux: low-water period 10^9^ molCO_2_
Carbonic acid	Carbonate	CCW	284.24	124.89	100.37	44.10
	Limestone	CLW	282.64	118.72	99.81	41.92
	Dolomite	CDW	1.6	6.17	0.57	2.18
	Silicate	CSW	58.07	25.32	20.51	8.94
Sulfuric acid	Carbonate	SCW	43.61	22.19	15.40	7.83
	Limestone	SLW	43.37	21.09	14.32	7.45
	Dolomite	SDW	0.25	1.10	1.08	0.39
Total intensity or flux	–		385.92	172.40	105.48	45.21
Total	–		558.32		150.69	

The contribution from each end-member slightly varied in strength and flux at different times. (1) The SCW flux during the high- and low-water periods was 15.40 × 10^9^ and 7.83 × 10^9^ mol CO_2_, respectively, accounting for 11.3% and 12.9% of the total flux from rock weathering. (2) The CO_2_ flux consumed by CSW during the high- and low-water periods accounted for 15.1% and 14.7% of the total CO_2_ flux from rock weathering, respectively. (3) The CO_2_ flux consumed by CCW during the high-water period accounted for 73.7% of the total CO_2_ flux of rock weathering, which decreased to 72.5% during the low-water period. In general, changes in the weathering of the carbonate and silicate end-members were not evident, whereas they were highly noticeable for the limestone and dolomite end-members. During the high-water period, the flux of CO_2_ consumed by CLW was 99.81 × 10^9^ mol, accounting for 73.2% of the total CO_2_ flux from rock weathering. In the low-water period, the flux was 41.92 × 10^9^ mol, accounting for only 68.9% of the total flux. The contribution from the dolomite end-member from carbonation to the CO_2_ flux increased from 0.4% during the high-water period to 3.6% in the low-water period. The total CO_2_ flux generated by limestone weathering during the high- and low-water periods was 10.5% and 12.2%, respectively, while the total CO_2_ flux due to dolomite varied from 0.8 to 0.6%, reflecting the variable weathering of limestone and dolomite at different times. For the Xijiang River Basin, carbonation was the main factor that affected weathering. During the high-water period, higher water temperatures had an inhibitory effect on the weathering and dissolution of limestone and dolomite, which yielded similar weathering rates. The dolomite weathering rate, however, was slightly elevated compared with the limestone weathering rate. Due to its large area in the basin, limestone became the main end-member for CO_2_ consumption via chemical weathering. During the low-water period, when the water temperature was low, the dolomite weathering rate was higher than that of limestone, resulting in an increase in the CO_2_ consumption due to dolomite weathering. In addition, the CO_2_ flux consumed by the carbonated weathered limestone end-member (CLW) decreased from 73.7% in the high-water period to 72.5% in the low-water period, i.e., a decrease of 1.2%. However, for the carbonated weathered dolomite end-member (CDW), this increase was 3.2%. This difference of 2.0% indicates that the weathering of limestone and dolomite were not completely complementary to each other. This is exemplified by the difference in the Ca/Mg ratios between the high- and low-water periods at point XJ18. During the high-water period, the weathering of limestone and dolomite tended to be similar. The weathering of large areas of limestone provides more Ca^2+^. More anthropogenic activity in the high-water period promoted the weathering of rocks, further strengthening the weathering of dolomite, which resulted in a lower Ca/Mg ratio. During the low-water period, there was an increase in the weathering of limestone and dolomite while the effect of anthropogenic activity on rock weathering was relatively low. Large-scale limestone weathering became the main source of solutes at the study sites, increasing the Ca/Mg ratio. As the main weathering end-member, the characteristics of limestone yielded consistent Sr isotope ratios during both the high- and low-water periods. The strengthened dolomite weathering during the low-water period produced Sr isotope ratios slightly more radiogenic than those in the high-water period. Finally, assuming that the runoff was the same between the high- and low-water periods, our calculations show that the limestone weathering strength and consumed CO_2_ flux were not significantly different between the two periods, whereas there was a larger increase in the dolomite weathering intensity and consumed CO_2_ flux. This situation fully demonstrates that both structural and random factors strongly effected dolomite weathering. Therefore, analyses of the material source throughout the Xijiang River Basin require not only an accurate account of the rock distribution but also full consideration of the weathering characteristics of the rocks themselves, as well as the role of anthropogenic activity.

Comparing the results listed in Table 11 with previous studies^6,35,36,39,60^, the total CO_2_ flux and fluxes from CCW and CSW are within the same order of magnitude. Except for Gaillardet et al.^39^, the total flux was not significantly different from other studies^6,35,36,60^. We verified that flow is the major factor in the flux calculation. Xu and Liu^60^ used the forward model and mass conservation for their calculations, whose results are consistent with the results of this study. However, due to the selection of the initial end-members in different regions^49^ (such as (Ca + Mg)/Na = 0.6), the forward model calculation process easily yields a negative calcium carbonate source. In addition, the atmospheric Cl^−^ input was equal to the lowest concentration obtained in this study. The source of Cl^−^ in the river may also be less than the summed input from anthropogenic activities, atmospheric input, and evaporite, which may eventually lead to non-conservation of mass, yielding an increase in the systematic error. The carbon fluxes generated by the CCW and CSW differed from previous studies^6,35,36,39,60^. The CCW value was similar to that of Sun et al.^36^ but less than those in the studies listed in Table 11. The reason for this may be that the combination of high-resolution and hydrogeochemical methods reduces the uncertainty caused by flooding or anthropogenic activities. In addition, we distinguished the dolomite and limestone end-members, which reduced the error to a certain extent. Moon et al.^41^ used the inverse model and bootstrap method to recalculate the silicate weathering rates for major global rivers based on Gaillardet et al.^39^, obtaining results similar to this study. However, there were slight differences. First, for a wide range of estimates, the method used in Moon et al.^41^ allows the calculation of the global source for large rivers in the absence of Sr isotope data. In a small range, especially for increased anthropogenic activities, there are more uncertainties in the Sr isotope data. Therefore, we must consider the corresponding relationship between the Sr isotope and “dynamic environment.” Moreover, due to the different sampling scales in time and space, there are differences in the representativeness of the samples. This was the original intent of the spatial structure (i.e., structural and random factors) proposed in this study. The carbon flux produced by the CSW is similar to that of Gaillardet et al.^39^. This may be due to the fact that both studies are based on inversion models, i.e., a substantial consistency in the calculation of the sulfuric acid source. However, the carbon flux produced by the CSW was only approximately one-sixth to one-half of those reported in previous studies^6,35,36,39,60^, which may be due to the two following reasons. (1) This study focused on the differences in the weathering and dissolution of rocks at different time intervals, qualitatively and quantitatively analyzing carbonated silicate rocks using inversion models. (2) The flow during the high-water period was large and the CCW intensity increased due to multiple floods, which caused a decrease in the CSW strength.

**Table 11:** A comparison of the results of this study with previous studies.

Catchment area (10^4^ km^2^)	Flow (km^3^/year)	Absorption of CO_2_ flux by CCW (10^6^ t/year)	Absorption of CO_2_ flux by CSW (10^6^ t/year)	References
35.3	200	6.4	1.0	This study
32.7	215	6.8 ± 0.3	2.4 ± 0.3	Zhong et al.^6^
35.2	230	12.5	2.4	Xu and Liu^60^
35.2	218.1	13.0	6.1	Sun et al.^36^
35.3	230	18.5	5.3	Gao et al.^35^
43.7	363	12.5	1.1	Gaillardet et al.^39^

## Conclusions

A multi-model combination and classical hydrogeochemical method were used in this study to estimate the carbon sink flux and ratios of each end-member for the Xijiang River Basin at monthly and annual scales.

1. Using the semi-variance model of Sr and its isotopes, the structural factors were the main control of Sr and its isotopes during the high-water period. In the low-water period, random and structural factors together controlled Sr and its isotopes. The random factors had a weaker impact during the high-water period than during the low-water period.
2. Using the path model analysis, we refined the end-members of river weathering and found the causal relationship between each end-member and Sr and its isotopes. Silicate weathering plays a leading role in determining the Sr and its isotopes during the high-water period. However, during the low-water period, dolomite weathering dissolution was dominant. Moreover, random factors during the low-water period had effects in the same direction as the structural factors. The influence of the random factors was much smaller than that of the structural factors such that the former can be ignored.
3. Using the inversion model, the material contribution from each end-member was quantified. In the Xijiang River Basin, the total dissolved substances were mainly derived from carbonate weathering, which was approximately 76% (0–96%) whereas silicate weathering accounted for only 14% (5–19%). The inversion model was used to estimate the optimum silicate weathering rate of 7.264–35.551 × 10^3^ mol/km^2^/year, where carbonic acid was the main factor that induces weathering. During the study period, the flux of CO_2_ in the atmosphere consumed by rock weathering was 150.69 × 10^9^ mol/year, which was 105.48 and 45.21 × 10^9^ mol CO_2_/year for the high- and low-water periods (66 and 34% of the total flux), respectively. The CO_2_ fluxes of CCW and CSW were 144.47 and 29.45 × 10^9^ mol CO_2_/year, respectively, and that of SCW was 23.23 × 10^9^ mol CO_2_/year. Compared with previous studies, the total carbon flux and those of CCW and CSW were all within the same order of magnitude.
4. Nevertheless, there were several differences between this study and previous studies such that the methods may require improvements. First, model applicability to the study area should be verified before performing calculations. Second, there were large differences in the selected range for the model-related parameters. As the selection of parameters has a significant influence on the final calculation results, the actual parameters applicable only to the specific study area should be used. Finally, actual base monitoring data, sampling frequency, spatial distribution density, and monitoring frequency affect the accuracy of the final model calculation results. Therefore, future studies should use high-precision monitoring and automatic sampling combined with improved mathematical models adapted to a specific study area.

## Supplementary Information

Supplementary Tables.
