# Supplementary material for: New estimate of chemical weathering rate in Xijiang River Basin based on multi-model
Source: Sci Rep. 2021 Mar 11;11:5728. doi: 10.1038/s41598-021-84602-1 (PMC7952734; doi:10.1038/s41598-021-84602-1)
Supplement: Supplementary file 1 — Supplementary Tables. [file 41598_2021_84602_MOESM1_ESM.docx]

# Chemical weathering and CO_2_ consumption in the Xijiang River Basin, China: carbonate and silicate weathering and anthropogenic impacts

Yong Zhang^①^ ^a, b^ , Shi Yu^①^ ^b*^, Shiyi He ^b^ , Pingan Sun ^b^ , Fu Wu ^a^, Zhenyu Liu ^a^, Haiyan Zhu ^c^, Xiao Li ^b,e^, Peng Zeng ^b,d^

^a^ Guangxi Geoenvironmental Monitoring Station, 530029, Nanning, Guangxi, China

^b^ Institute of Karst Geology CAGS/Key Laboratory of Karst Dynamics, MNR & GZAR. Guilin, Guangxi, 541004, China

^c^ Guangxi Branch of China National Geological Explration Center of Building Material Industry, Guilin, Guangxi, 541004, China

^d^ College of Geography and Environmental Science, Northwest Normal University, 730070, Lanzhou, China

^e^ College of Environmental Science and Engineering, Guilin University of Technology, 514006, Guilin China

^①^ **First author. Yong Zhang and Shi Yu**

***Corresponding author.** **E-mail: yushi@karst.ac.cn (Shi Yu)**

**The hydrochemical data of river in low water period**

| Sample NO | **T(℃)** | **Arae (km^2^)** | **Discharge（km^3^/yr）** | **pH** | **K^+^ (mg/L)** | **Na^+^ (mg/L)** | **Ca^2+^ (mg/L)** | **Mg^2+^ (mg/L)** | **Cl^-^(mg/L)** | **SO_4_^2-^(mg/L)** | **HCO_3_^-^（mg/L）** | **NO_3_^-^(mg/L)** | **Sr(mg/L)** | **SiO_2_ (mg/L）** | **TDS（mg/L）** | **^87^Sr/^86^Sr** |
| --- | --- | --- | --- | --- | --- | --- | --- | --- | --- | --- | --- | --- | --- | --- | --- | --- |
|  |  |  |  |  |  |  |  |  |  |  |  |  |  |  |  |  |
|  |  |  |  |  |  |  |  |  |  |  |  |  |  |  |  |  |
| XJ01 | 17.41 | 4364.18 | 17.47 | 7.95 | 1.56 | 3.69 | 57.22 | 8.26 | 3.37 | 32.12 | 81.88 | 8.89 | 0.247 | 3.92 | 188.1 | 0.708426 |
| XJ02 | 17.69 | 4088 | 8.83 | 8.04 | 1.54 | 3.74 | 58.34 | 8.5 | 3.34 | 30.97 | 174.44 | 5.89 | 0.229 | 4.02 | 280.87 | 0.708449 |
| XJ03 | 17.73 | 112500 | 12.74 | 7.74 | 1.55 | 3.72 | 56.67 | 8.53 | 3.31 | 30.8 | 174.44 | 8.34 | 0.213 | 2.43 | 279.02 | 0.708454 |
| XJ04 | 18.16 | 112200 | 125.40 | 8.05 | 1.54 | 3.71 | 56.41 | 8.54 | 3.28 | 30.94 | 176.22 | 8.19 | 0.23 | 4.12 | 280.64 | 0.708451 |
| XJ05 | 17.74 | 106580 | 111.00 | 7.91 | 1.58 | 3.78 | 56.82 | 8.64 | 3.34 | 31.54 | 178 | 8.6 | 0.248 | 1.05 | 283.7 | 0.708457 |
| XJ06 | 17.35 | 98500 | 103.40 | 8.07 | 1.63 | 4.01 | 56.24 | 8.96 | 3.44 | 32.77 | 172.66 | 6.98 | 0.267 | 3.57 | 279.71 | 0.708456 |
| XJ07 | 17.28 | 3196 | 2.67 | 7.83 | 1.69 | 4.11 | 57.25 | 9.27 | 3.58 | 33.92 | 177.11 | 8.8 | 0.261 | 3.67 | 286.93 | 0.70844 |
| XJ08 | 14.76 | 3273 | 2.68 | 7.97 | 1.7 | 3.53 | 32.86 | 4.06 | 4.46 | 11.29 | 106.8 | 6.64 | 0.0534 | 2.58 | 164.7 | 0.712445 |
| XJ09 | 12.94 | 2680 | 9.84 | 7.75 | 1.88 | 2.81 | 45.39 | 7.32 | 5.44 | 14.45 | 149.52 | 6.8 | 0.0534 | 1.74 | 226.81 | 0.710637 |
| XJ10 | 12.74 | 6380 | 448.00 | 7.88 | 1.93 | 4.02 | 29.28 | 4.46 | 6.6 | 11.6 | 99.68 | 6.83 | 0.0514 | 3.23 | 157.57 | 0.712348 |
| XJ11 | 13.58 | 2989 | 101.55 | 7.88 | 1.77 | 3.38 | 31.19 | 4.04 | 4.39 | 11.17 | 103.24 | 2.29 | 0.0568 | 3.32 | 159.18 | 0.712763 |
| XJ12 | 13.54 | 10606 | 57.07 | 7.81 | 2 | 4.12 | 41.43 | 5.5 | 4.8 | 19.78 | 135.28 | <0.05 | 0.121 | 4.91 | 212.91 | 0.709798 |
| XJ13 | 15.92 | 22112 | 8.77 | 8.00 | 1.97 | 4.48 | 48.19 | 6.71 | 5.21 | 23.85 | 153.08 | 0.01 | 0.156 | 4.71 | 243.49 | 0.708934 |
| XJ14 | 17.27 | 1966.55 | 8.79 | 7.90 | 1.67 | 3.85 | 50.08 | 7.16 | 3.94 | 2.64 | 174.44 | 8.52 | 0.21 | 4.22 | 243.78 | 0.708632 |
| XJ15 | 17.56 | 8633.3 | 3.80 | 7.82 | 2.61 | 6.97 | 51.84 | 4.82 | 8.67 | 14.15 | 167.32 | <0.05 | 0.0685 | 5.55 | 256.38 | 0.710603 |
| XJ16 | 18.85 | 7265.6 | 4.24 | 7.77 | 1.82 | 3.77 | 50.1 | 4.96 | 4.7 | 13.99 | 163.76 | 6.91 | 0.0817 | 4.86 | 243.1 | 0.710054 |
| XJ17 | 17.53 | 11855 | 13.56 | 7.68 | 1.82 | 4.06 | 44.83 | 4.35 | 5.03 | 10.37 | 142.4 | 2.01 | 0.0609 | 6.44 | 212.86 | 0.711155 |
| XJ18 | 17.89 | 2682.3 | 0.74 | 7.81 | 1.44 | 3.23 | 39.87 | 4.4 | 3.48 | 2.64 | 142.4 | 5.77 | 0.0603 | 6 | 197.46 | 0.71157 |
| XJ19 | 17.2 | 4281 | 1.49 | 7.86 | 1.55 | 3.57 | 37.93 | 4 | 2.84 | 7.45 | 135.28 | 4.44 | 0.0696 | 7.78 | 192.62 | 0.710364 |
| XJ20 | 17.12 | 1213.81 | 0.83 | 7.88 | 1.04 | 2.73 | 58.77 | 5.83 | 2.93 | 2.64 | 188.68 | 10.97 | 0.0594 | 3.87 | 262.62 | 0.710248 |

**The hydrochemical data of river in high water period**

| Sample NO | **T(℃)** | **Arae (km^2^)** | **Discharge（km^3^/yr）** | **pH** | **K^+^ (mg/L)** | **Na^+^ (mg/L)** | **Ca^2+^ (mg/L)** | **Mg^2+^ (mg/L)** | **Cl^-^(mg/L)** | **SO_4_^2-^(mg/L)** | **HCO_3_^-^（mg/L）** | **NO_3_^-^(mg/L)** | **Sr(mg/L)** | **SiO_2_ (mg/L）** | **TDS（mg/L）** | **^87^Sr/^86^Sr** |
| --- | --- | --- | --- | --- | --- | --- | --- | --- | --- | --- | --- | --- | --- | --- | --- | --- |
|  |  |  |  |  |  |  |  |  |  |  |  |  |  |  |  |  |
|  |  |  |  |  |  |  |  |  |  |  |  |  |  |  |  |  |
| XJ01 | 24.47 | 4364.18 | 87.35 | 7.83 | 1.24 | 2.65 | 57.65 | 6.11 | 3.23 | 12.37 | 177.85 | 6.82 | 0.17 | 5.82 | 266.92 | 0.709909 |
| XJ02 | 24.44 | 4088 | 44.15 | 7.83 | 1.34 | 3.04 | 60.32 | 6.67 | 4.05 | 24.13 | 187.42 | 8.87 | 0.17 | 5.65 | 292.62 | 0.708452 |
| XJ03 | 23.69 | 112500 | 63.70 | 7.76 | 1.32 | 3.1 | 60.72 | 6.88 | 4.1 | 27.18 | 187.42 | 8.18 | 0.18 | 6.1 | 296.82 | 0.708453 |
| XJ04 | 24 | 112200 | 627.00 | 7.81 | 1.4 | 3.51 | 59.4 | 7.45 | 3.56 | 19.94 | 183.59 | 7.01 | 0.2 | 5.09 | 283.94 | 0.708454 |
| XJ05 | 28.63 | 106580 | 555.00 | 8.55 | 1.21 | 2.74 | 45.72 | 5.18 | 2.89 | 13.24 | 133.87 | 10.2 | 0.14 | 4.3 | 209.15 | 0.708457 |
| XJ06 | 29.58 | 98500 | 517.00 | 8.78 | 1.64 | 4.73 | 36.29 | 9.08 | 4.76 | 35.95 | 118.57 | 6.68 | 0.24 | 2.06 | 213.08 | 0.708448 |
| XJ07 | 24.24 | 3196 | 13.34 | 7.77 | 1.81 | 5.32 | 59.44 | 10.69 | 5.4 | 39.96 | 175.94 | 8.28 | 0.28 | 5.37 | 303.93 | 0.708902 |
| XJ08 | 28.3 | 3273 | 13.40 | 8.4 | 1.03 | 1.57 | 27.71 | 3.42 | 3.05 | 10.09 | 87.97 | <0.05 | 0.044 | 7.45 | 142.29 | 0.711541 |
| XJ09 | 25.8 | 2680 | 49.20 | 8.44 | 1.56 | 2.95 | 43.58 | 6.12 | 6.7 | 15.26 | 145.34 | <0.05 | 0.062 | 6.33 | 227.84 | 0.711493 |
| XJ10 | 27.5 | 6380 | 2240.00 | 8.36 | 1.26 | 2.21 | 21.87 | 3.28 | 3.62 | 10.23 | 72.67 | 6.67 | 0.04 | 9.82 | 124.96 | 0.711459 |
| XJ11 | 26.8 | 2989 | 507.73 | 8.31 | 0.92 | 1.37 | 25.79 | 3.16 | 2.93 | 9.7 | 84.15 | 4.73 | 0.04 | 7.62 | 135.64 | 0.710887 |
| XJ12 | 27.1 | 10606 | 285.34 | 8.23 | 1.15 | 1.97 | 29.42 | 3.65 | 3.53 | 11.92 | 87.97 | <0.05 | 0.06 | 8.52 | 148.13 | 0.712861 |
| XJ13 | 27.1 | 22112 | 43.84 | 7.91 | 1.54 | 2.2 | 34.38 | 3.97 | 4.65 | 14.4 | 103.27 | <0.05 | 0.083 | 8.18 | 172.59 | 0.708783 |
| XJ14 | 25.5 | 1966.55 | 43.96 | 8.28 | 1.22 | 2.28 | 36.82 | 4.42 | 4.14 | 16.22 | 107.09 | <0.05 | 0.1 | 8.07 | 180.26 | 0.709618 |
| XJ15 | 28.7 | 8633.3 | 19.02 | 8.23 | 2.34 | 3.9 | 51.94 | 5.36 | 8.49 | 16.56 | 160.64 | <0.05 | 0.083 | 8.8 | 258.03 | 0.710329 |
| XJ16 | 28.1 | 7265.6 | 21.19 | 8.2 | 1.81 | 2.59 | 56.27 | 5.07 | 6.36 | 15.52 | 166.38 | <0.05 | 0.087 | 8.13 | 262.13 | 0.710605 |
| XJ17 | 27.8 | 11855 | 67.80 | 8.25 | 1.02 | 1.61 | 59.86 | 4.7 | 4.4 | 13.7 | 179.77 | 8.12 | 0.076 | 7.96 | 273.02 | 0.711363 |
| XJ18 | 28.8 | 2682.3 | 3.69 | 8.34 | 1.53 | 1.85 | 8.22 | 4.16 | 4.87 | 12.36 | 145.34 | 9.75 | 0.078 | 8.35 | 186.68 | 0.711047 |
| XJ19 | 29 | 4281 | 7.44 | 8.53 | 1.16 | 1.89 | 48.1 | 4.42 | 3.12 | 10.44 | 156.82 | 4.38 | 0.08 | 9.7 | 235.65 | 0.710306 |
| XJ20 | 28 | 1213.81 | 4.16 | 8.56 | 0.9 | 2.05 | 61.54 | 5.19 | 3.64 | 23.93 | 183.59 | 8.37 | 0.075 | 6.16 | 287 | 0.710334 |

**The parameters of each endmember of the model**

| End member | Ca/Na | Mg/Na | HCO_3_/Na | Cl/Na | 1000*Sr/Na | ^87^Sr/^86^Sr |
| --- | --- | --- | --- | --- | --- | --- |
| Rain |  |  |  |  |  |  |
| High water period | 3.83 | 1.08 | 23.14 | 1.41 | 16.51 | 0.709 |
| Low-water period | 1.66 | 0.3 | 13.58 | 0.61 | 9.69 | 0.709 |
| Carbonate |  |  |  |  |  |  |
| High water period | 70 | 12.18 | 137.95 | 0.001 | 50 | 0.7088 |
| Low-water period | 69.32 | 17.56 | 132.34 | 0.001 | 50 | 0.7087 |
| Silicate |  |  |  |  |  |  |
| High water period | 0.56 | 0.62 | 1.17 | 0.001 | 3.06 | 0.791 |
| Low-water period | 0.56 | 0.39 | 1 | 0.001 | 10.06 | 0.7804 |

**The data of flow**

| Time | 2014.01 | 2014.02 | 2014.03 | 2014.04 | 2014.05 | 2014.06 | 2014.07 | 2014.08 | 2014.09 | 2014.10 | 2014.11 | 2014.12 |
| --- | --- | --- | --- | --- | --- | --- | --- | --- | --- | --- | --- | --- |
| Q（m^3^/s） | 2094.28 | 1647.60 | 2444.45 | 7542.61 | 9898.21 | 12605.5 | 11996.9 | 9533.26 | 7700.36 | 4468.44 | 4182.54 | 2044.4 |

**Sampling point location**

| Sample NO | ID | N | E |
| --- | --- | --- | --- |
| XJ01 | 1 | 23°37.434′ | 108°57.581′ |
| XJ02 | 2 | 23°50.342′ | 108°09.324′ |
| XJ03 | 3 | 23°50.820′ | 108°07.837′ |
| XJ04 | 4 | 23°43.479′ | 107°58.659′ |
| XJ05 | 5 | 24°02.491′ | 107°31.084′ |
| XJ06 | 6 | 25°01.490′ | 107°02.498′ |
| XJ07 | 7 | 25°00.958′ | 107°34.341′ |
| XJ08 | 8 | 24°10'10.8'' | 110°49'26.3'' |
| XJ09 | 9 | 24°23'52.3'' | 110°33'55.3'' |
| XJ10 | 10 | 23°59'25.7'' | 111°43'50.6'' |
| XJ11 | 11 | 23°43'19.0'' | 111°02'50.8'' |
| XJ12 | 12 | 23°26'38.2'' | 111°29'43.0'' |
| XJ13 | 13 | 23°31'39.4'' | 110°24'21.5'' |
| XJ14 | 14 | 23°35'59.0'' | 109°39'07.0'' |
| XJ15 | 15 | 23°05'01.5'' | 109°36'10.0'' |
| XJ16 | 16 | 22°49'40.9'' | 108°15'09.8'' |
| XJ17 | 17 | 22°39'43.6'' | 107°54'15.5'' |
| XJ18 | 18 | 22°24'34.2'' | 107°21'13.5'' |
| XJ19 | 19 | 22°19'56.2'' | 106°52'15.9'' |
| XJ20 | 20 | 22°44'31.3'' | 106°59'49.6'' |

**Rain water chemistry and related parameters in Guilin city(table a, b and c are yaoshan campus of Guilin university of electronic science and technology, Guilin environmental monitoring station and Longyinlong primary school，respectively)**

**(a) Yaoshan campus of Guilin university of electronic science and technology**

| Date | Rain | Ec | SO_4_^2-^ | NO_3_^-^ | F^-^ | Cl^-^ | NH_4_^+^ | Ca^2+^ | Mg^2+^ | Na^+^ | K^+^ |
| --- | --- | --- | --- | --- | --- | --- | --- | --- | --- | --- | --- |
|  | (mm) | (ms/m) | (mg/L) | (mg/L) | (mg/L) | (mg/L) | (mg/L) | (mg/L) | (mg/L) | (mg/L) | (mg/L) |
| 2016/1/5 | 12.60 | 2.30 | 4.47 | 1.75 | 0.11 | 0.73 | 0.68 | 1.36 | 0.21 | 0.77 | 0.25 |
| 2016/1/8 | 15.30 | 4.17 | 3.97 | 2.68 | 0.28 | 0.49 | 1.00 | 0.66 | 0.06 | 0.23 | 0.22 |
| 2016/1/9 | 14.20 | 3.33 | 3.53 | 1.89 | 0.11 | 0.36 | 0.74 | 0.48 | 0.05 | 0.13 | 0.18 |
| 2016/1/14 | 5.30 | 2.26 | 3.97 | 1.91 | 0.20 | 0.46 | 0.58 | 1.19 | 0.14 | 0.33 | 0.24 |
| 2016/1/15 | 9.20 | 1.75 | 2.96 | 1.21 | 0.08 | 0.22 | 0.32 | 0.73 | 0.05 | 0.24 | 0.16 |
| 2016/1/28 | 5.80 | 2.00 | 2.82 | 0.91 | 0.07 | 1.08 | 0.10 | 1.64 | 0.35 | 0.70 | 0.24 |
| 2016/1/31 | 11.00 | 1.59 | 1.94 | 1.50 | 0.03 | 0.25 | 0.45 | 0.66 | 0.08 | 0.17 | 0.17 |
| 2016/2/1 | 15.00 | 1.01 | 1.15 | 1.06 | 0.04 | 0.23 | 0.20 | 0.58 | 0.09 | 0.09 | 0.16 |
| 2016/3/9 | 18.60 | 2.67 | 4.95 | 3.85 | 0.07 | 0.75 | 1.67 | 1.32 | 0.33 | 0.35 | 0.36 |
| 2016/3/10 | 28.30 | 3.72 | 8.61 | 3.14 | 0.12 | 1.35 | 2.50 | 1.97 | 0.49 | 0.70 | 0.29 |
| 2016/3/17 | 22.40 | 2.76 | 4.68 | 2.70 | 0.12 | 0.98 | 0.90 | 1.94 | 0.40 | 0.29 | 0.25 |
| 2016/3/19 | 22.70 | 4.20 | 6.20 | 4.07 | 0.14 | 1.24 | 1.57 | 2.02 | 0.58 | 0.45 | 0.23 |
| 2016/3/20 | 113.00 | 2.47 | 3.76 | 2.35 | 0.07 | 0.29 | 1.22 | 0.86 | 0.16 | 0.13 | 0.29 |
| 2016/3/21 | 30.50 | 3.62 | 7.11 | 4.18 | 0.08 | 0.83 | 1.89 | 1.69 | 0.40 | 0.33 | 0.29 |
| 2016/3/22 | 42.30 | 1.77 | 2.05 | 1.45 | 0.25 | 0.28 | 0.62 | 0.34 | 0.09 | 0.10 | 0.13 |
| 2016/4/2 | 33.20 | 3.70 | 5.76 | 2.90 | 0.08 | 1.14 | 1.45 | 1.66 | 0.45 | 0.72 | 0.36 |
| 2016/4/3 | 58.30 | 1.00 | 1.24 | 0.66 | 0.13 | 0.19 | 0.17 | 0.76 | 0.11 | 0.17 | 0.12 |
| 2016/4/5 | 8.30 | 4.73 | 5.21 | 3.65 | 0.10 | 1.58 | 1.77 | 3.17 | 0.47 | 0.75 | 0.25 |
| 2016/4/8 | 36.80 | 2.15 | 3.50 | 2.00 | 0.07 | 0.41 | 1.17 | 0.75 | 0.12 | 0.02 | 0.21 |
| 2016/4/9 | 36.50 | 1.22 | 1.83 | 0.81 | 0.05 | 0.13 | 0.37 | 0.38 | 0.10 | 0.17 | 0.14 |
| 2016/4/10 | 36.70 | 1.33 | 2.12 | 1.18 | 0.05 | 0.20 | 0.44 | 0.77 | 0.10 | 0.16 | 0.10 |
| 2016/4/11 | 36.70 | 1.67 | 2.81 | 1.50 | 0.10 | 0.29 | 0.67 | 0.81 | 0.17 | 0.31 | 0.14 |
| 2016/4/12 | 34.20 | 1.82 | 2.66 | 1.67 | 0.20 | 0.32 | 0.87 | 0.70 | 0.13 | 0.32 | 0.19 |
| 2016/4/15 | 13.60 | 2.03 | 2.63 | 1.72 | 0.16 | 0.71 | 0.00 | 1.42 | 0.32 | 0.60 | 0.23 |
| 2016/4/16 | 23.40 | 2.28 | 3.58 | 1.94 | 0.09 | 0.51 | 0.71 | 1.09 | 0.21 | 0.34 | 0.23 |
| 2016/4/17 | 38.10 | 2.65 | 2.79 | 2.43 | 0.06 | 0.30 | 0.94 | 0.43 | 0.09 | 0.22 | 0.16 |
| 2016/4/19 | 13.60 | 1.74 | 2.99 | 2.26 | 0.11 | 0.57 | 0.58 | 2.27 | 0.20 | 0.24 | 0.11 |
| 2016/4/21 | 36.80 | 1.58 | 2.39 | 1.16 | 0.08 | 0.29 | 0.41 | 0.70 | 0.15 | 0.27 | 0.00 |
| 2016/4/24 | 11.50 | 2.06 | 4.14 | 1.86 | 0.13 | 0.44 | 0.89 | 1.06 | 0.32 | 0.31 | 0.14 |
| 2016/4/28 | 32.20 | 1.37 | 1.88 | 0.86 | 0.12 | 0.17 | 0.42 | 0.23 | 0.07 | 0.32 | 0.10 |
| 2016/4/29 | 32.20 | 2.50 | 6.27 | 2.42 | 0.08 | 0.43 | 1.35 | 0.74 | 0.17 | 0.30 | 0.21 |
| 2016/4/30 | 4.80 | 3.70 | 7.44 | 3.09 | 0.08 | 0.87 | 1.51 | 1.65 | 0.40 | 0.50 | 0.32 |
| 2016/5/4 | 37.70 | 1.94 | 2.43 | 1.63 | 0.06 | 0.23 | 0.86 | 0.40 | 0.08 | 0.21 | 0.16 |
| 2016/5/5 | 32.20 | 0.60 | 0.88 | 0.70 | 0.05 | 0.09 | 0.23 | 0.22 | 0.07 | 0.10 | 0.11 |
| 2016/5/7 | 34.20 | 1.63 | 2.42 | 1.59 | 0.11 | 0.31 | 0.82 | 0.47 | 0.12 | 0.19 | 0.16 |
| 2016/5/8 | 27.80 | 1.46 | 2.11 | 1.32 | 0.06 | 0.28 | 0.57 | 0.47 | 0.13 | 0.21 | 0.15 |
| 2016/5/11 | 36.80 | 0.73 | 1.14 | 0.43 | 0.04 | 0.15 | 0.27 | 0.23 | 0.10 | 0.09 | 0.10 |
| 2016/5/13 | 36.70 | 2.75 | 4.11 | 2.27 | 0.08 | 0.56 | 1.09 | 0.72 | 0.22 | 0.32 | 0.21 |
| 2016/5/15 | 36.50 | 1.03 | 1.47 | 0.82 | 0.10 | 0.31 | 0.02 | 0.75 | 0.18 | 0.22 | 0.08 |
| 2016/5/18 | 34.60 | 3.22 | 6.67 | 2.93 | 0.07 | 0.56 | 1.51 | 1.07 | 0.24 | 0.30 | 0.19 |
| 2016/5/19 | 45.20 | 1.74 | 2.46 | 1.17 | 0.10 | 0.32 | 0.47 | 0.54 | 0.14 | 0.14 | 0.12 |
| 2016/5/25 | 32.70 | 1.01 | 1.98 | 0.55 | 0.06 | 0.17 | 0.34 | 0.29 | 0.12 | 0.22 | 0.10 |
| 2016/5/29 | 12.80 | 1.98 | 2.02 | 1.43 | 0.17 | 0.49 | 0.32 | 0.23 | 0.30 | 0.23 | 0.14 |
| 2016/5/30 | 26.50 | 1.61 | 2.67 | 0.69 | 0.05 | 0.45 | 0.29 | 0.24 | 0.27 | 0.24 | 0.00 |
| 2016/6/3 | 35.60 | 0.52 | 0.82 | 0.33 | 0.04 | 0.07 | 0.08 | 0.19 | 0.06 | 0.05 | 0.07 |
| 2016/6/4 | 36.10 | 0.66 | 0.96 | 0.34 | 0.05 | 0.09 | 0.10 | 0.19 | 0.08 | 0.09 | 0.07 |
| 2016/6/7 | 35.20 | 1.86 | 3.04 | 1.42 | 0.14 | 0.31 | 0.74 | 0.91 | 0.20 | 0.19 | 0.12 |
| 2016/6/8 | 32.00 | 1.77 | 2.66 | 0.93 | 0.05 | 0.24 | 0.65 | 0.42 | 0.17 | 0.10 | 0.08 |
| 2016/6/9 | 17.60 | 2.72 | 3.75 | 1.00 | 0.72 | 0.87 | 0.65 | 1.06 | 0.51 | 0.48 | 0.13 |
| 2016/6/11 | 31.50 | 1.14 | 1.43 | 0.53 | 0.10 | 0.20 | 0.15 | 0.38 | 0.16 | 0.11 | 0.10 |
| 2016/6/12 | 36.80 | 0.85 | 1.21 | 0.31 | 0.04 | 0.08 | 0.11 | 0.24 | 0.08 | 0.06 | 0.07 |
| 2016/6/13 | 36.40 | 0.86 | 0.84 | 0.74 | 0.08 | 0.19 | 0.12 | 0.25 | 0.10 | 0.13 | 0.07 |
| 2016/6/15 | 45.20 | 0.48 | 0.66 | 0.50 | 0.06 | 0.09 | 0.21 | 0.15 | 0.06 | 0.05 | 0.08 |
| 2016/6/30 | 33.60 | 1.55 | 1.17 | 1.43 | 0.29 | 0.72 | 0.00 | 0.76 | 0.38 | 0.48 | 0.10 |
| 2016/7/2 | 23.80 | 1.60 | 1.68 | 1.32 | 0.20 | 0.73 | 0.42 | 0.66 | 0.29 | 0.37 | 0.10 |
| 2016/7/3 | 13.70 | 1.37 | 1.22 | 1.15 | 0.31 | 0.66 | 0.17 | 0.75 | 0.26 | 0.36 | 0.09 |
| 2016/7/5 | 33.70 | 0.67 | 1.04 | 0.32 | 0.04 | 0.22 | 0.05 | 0.38 | 0.09 | 0.16 | 0.09 |
| 2016/7/6 | 33.70 | 1.00 | 1.96 | 0.54 | 0.13 | 0.38 | 0.00 | 0.79 | 0.19 | 0.18 | 0.09 |
| 2016/7/8 | 32.90 | 2.15 | 2.60 | 1.16 | 0.21 | 0.59 | 0.25 | 0.80 | 0.29 | 0.26 | 0.09 |
| 2016/7/11 | 33.70 | 1.50 | 2.02 | 1.05 | 0.13 | 0.38 | 0.56 | 0.53 | 0.16 | 0.18 | 0.11 |
| 2016/8/2 | 48.00 | 1.04 | 1.19 | 0.74 | 0.14 | 0.40 | 0.00 | 0.50 | 0.26 | 0.29 | 0.00 |
| 2016/8/3 | 41.00 | 1.94 | 1.82 | 1.57 | 0.30 | 1.16 | 0.06 | 0.65 | 0.59 | 0.82 | 0.14 |
| 2016/8/7 | 33.80 | 0.97 | 1.25 | 0.64 | 0.17 | 0.28 | 0.45 | 0.30 | 0.16 | 0.14 | 0.00 |
| 2016/8/10 | 33.60 | 2.45 | 3.81 | 1.14 | 0.05 | 0.39 | 0.50 | 0.88 | 0.26 | 0.17 | 0.12 |
| 2016/8/11 | 38.40 | 0.84 | 1.28 | 0.46 | 0.07 | 0.09 | 0.11 | 0.48 | 0.07 | 0.11 | 0.09 |
| 2016/8/14 | 15.20 | 3.34 | 3.09 | 1.47 | 0.53 | 1.36 | 0.01 | 1.55 | 0.52 | 0.70 | 0.11 |
| 2016/8/25 | 17.60 | 3.65 | 5.48 | 1.70 | 0.43 | 0.59 | 1.51 | 1.02 | 0.35 | 0.28 | 0.22 |
| 2016/10/18 | 14.70 | 3.56 | 3.74 | 3.26 | 0.95 | 1.79 | 0.50 | 1.70 | 1.05 | 0.92 | 0.25 |
| 2016/10/19 | 19.20 | 1.88 | 2.65 | 0.56 | 0.38 | 0.54 | 0.06 | 0.65 | 0.25 | 0.28 | 0.12 |
| 2016/10/25 | 17.60 | 2.88 | 4.42 | 2.83 | 0.17 | 1.35 | 0.07 | 1.48 | 0.69 | 0.69 | 0.08 |
| 2016/11/1 | 24.00 | 1.18 | 1.26 | 0.79 | 0.14 | 0.51 | 0.22 | 0.45 | 0.22 | 0.23 | 0.10 |
| 2016/11/10 | 33.40 | 0.78 | 1.16 | 0.41 | 0.16 | 0.31 | 0.12 | 0.42 | 0.12 | 0.21 | 0.13 |
| 2016/11/12 | 33.60 | 1.54 | 2.49 | 0.87 | 0.16 | 0.61 | 0.41 | 0.44 | 0.16 | 0.35 | 0.13 |
| 2016/11/13 | 32.50 | 2.37 | 3.60 | 1.11 | 0.14 | 0.70 | 0.29 | 0.64 | 0.28 | 0.35 | 0.09 |
| 2016/11/14 | 10.20 | 3.14 | 4.06 | 0.41 | 0.21 | 2.38 | 0.00 | 1.28 | 0.29 | 1.19 | 0.20 |
| 2016/11/21 | 11.20 | 1.42 | 2.42 | 0.85 | 0.19 | 0.46 | 0.34 | 0.62 | 0.21 | 0.21 | 0.00 |
| 2016/11/24 | 33.60 | 0.58 | 0.44 | 0.50 | 0.19 | 0.24 | 0.12 | 0.34 | 0.10 | 0.12 | 0.00 |
| 2016/11/26 | 10.20 | 1.08 | 1.17 | 0.88 | 0.27 | 0.38 | 0.03 | 0.50 | 0.18 | 0.21 | 0.26 |
| 2016/12/12 | 33.70 | 0.75 | 0.63 | 0.27 | 0.38 | 0.24 | 0.00 | 0.51 | 0.06 | 0.10 | 0.08 |
| 2016/12/19 | 24.00 | 3.33 | 5.66 | 3.83 | 0.11 | 1.36 | 0.77 | 3.02 | 0.21 | 0.58 | 0.17 |
| 2016/12/20 | 27.50 | 2.08 | 4.01 | 3.18 | 0.27 | 0.89 | 1.06 | 1.24 | 0.19 | 0.57 | 0.25 |
| 2016/12/21 | 24.30 | 2.52 | 3.59 | 2.44 | 0.39 | 0.76 | 0.93 | 1.08 | 0.22 | 0.45 | 0.22 |
| 2016/12/25 | 34.30 | 3.99 | 5.62 | 3.36 | 0.17 | 0.63 | 1.36 | 0.90 | 0.20 | 0.45 | 0.27 |

**(b) Guilin environmental monitoring station**

| Date | Rain | Ec | SO_4_^2-^ | NO_3_^-^ | F^-^ | Cl^-^ | NH_4_^+^ | Ca^2+^ | Mg^2+^ | Na^+^ | K^+^ |
| --- | --- | --- | --- | --- | --- | --- | --- | --- | --- | --- | --- |
|  | (mm) | (ms/m) | (mg/L) | (mg/L) | (mg/L) | (mg/L) | (mg/L) | (mg/L) | (mg/L) | (mg/L) | (mg/L) |
| 2016/1/5 | 27.20 | 2.37 | 3.68 | 1.54 | 0.09 | 0.26 | 0.66 | 0.78 | 0.08 | 0.27 | 0.20 |
| 2016/1/8 | 17.10 | 4.20 | 5.80 | 2.80 | 0.06 | 0.52 | 1.05 | 0.88 | 0.11 | 0.18 | 0.22 |
| 2016/1/9 | 16.40 | 3.92 | 5.29 | 2.30 | 0.12 | 0.42 | 0.99 | 0.53 | 0.06 | 0.21 | 0.20 |
| 2016/1/14 | 6.40 | 2.24 | 4.41 | 2.13 | 0.07 | 0.36 | 0.49 | 1.43 | 0.09 | 0.23 | 0.20 |
| 2016/1/15 | 8.60 | 1.84 | 3.21 | 1.28 | 0.07 | 0.32 | 0.50 | 0.76 | 0.08 | 0.19 | 0.22 |
| 2016/1/31 | 14.00 | 1.59 | 2.30 | 1.60 | 0.05 | 0.43 | 0.58 | 0.84 | 0.14 | 0.21 | 0.20 |
| 2016/2/1 | 15.00 | 4.03 | 4.70 | 5.00 | 0.08 | 2.16 | 0.90 | 2.44 | 0.47 | 2.18 | 0.32 |
| 2016/3/9 | 21.10 | 3.59 | 5.96 | 3.37 | 0.09 | 0.88 | 1.91 | 1.69 | 0.34 | 0.32 | 0.31 |
| 2016/3/10 | 21.00 | 3.61 | 6.74 | 3.38 | 0.05 | 0.63 | 1.72 | 1.33 | 0.33 | 0.22 | 0.30 |
| 2016/3/17 | 35.20 | 3.80 | 5.92 | 2.85 | 0.13 | 1.05 | 1.11 | 2.58 | 0.43 | 0.31 | 0.36 |
| 2016/3/19 | 23.00 | 4.16 | 6.71 | 3.75 | 0.14 | 1.62 | 0.88 | 2.49 | 0.41 | 0.68 | 0.31 |
| 2016/3/20 | 114.00 | 2.81 | 4.64 | 3.62 | 0.04 | 0.96 | 1.05 | 1.51 | 0.33 | 0.83 | 0.24 |
| 2016/3/21 | 37.20 | 3.46 | 6.43 | 3.20 | 0.08 | 0.74 | 1.25 | 1.92 | 0.39 | 0.43 | 0.28 |
| 2016/3/22 | 41.50 | 1.94 | 2.05 | 1.31 | 0.21 | 0.20 | 0.66 | 0.45 | 0.09 | 0.07 | 0.14 |
| 2016/4/3 | 61.00 | 0.68 | 0.72 | 0.46 | 0.20 | 0.11 | 0.21 | 0.23 | 0.09 | 0.20 | 0.12 |
| 2016/4/5 | 11.20 | 5.10 | 6.84 | 6.09 | 0.11 | 1.32 | 2.71 | 1.76 | 0.38 | 0.78 | 0.49 |
| 2016/4/8 | 24.00 | 3.03 | 5.34 | 3.48 | 0.10 | 0.71 | 2.10 | 0.86 | 0.14 | 0.57 | 0.33 |
| 2016/4/9 | 36.50 | 1.76 | 2.46 | 1.25 | 0.15 | 0.17 | 0.60 | 0.49 | 0.11 | 0.29 | 0.15 |
| 2016/4/10 | 36.90 | 1.08 | 1.71 | 0.87 | 0.11 | 0.11 | 0.22 | 0.64 | 0.07 | 0.19 | 0.09 |
| 2016/4/11 | 36.80 | 1.03 | 1.83 | 1.15 | 0.22 | 0.13 | 0.37 | 0.75 | 0.09 | 0.30 | 0.09 |
| 2016/4/12 | 33.60 | 2.51 | 4.16 | 2.17 | 0.13 | 0.34 | 1.21 | 0.79 | 0.14 | 0.42 | 0.22 |
| 2016/4/16 | 27.20 | 2.95 | 4.35 | 2.16 | 0.09 | 0.50 | 1.36 | 1.40 | 0.19 | 0.38 | 0.20 |
| 2016/4/17 | 36.70 | 2.30 | 2.59 | 2.27 | 0.06 | 0.27 | 0.89 | 0.49 | 0.10 | 0.17 | 0.22 |
| 2016/4/19 | 14.40 | 1.82 | 3.34 | 1.80 | 0.09 | 0.47 | 0.97 | 1.25 | 0.20 | 0.32 | 0.13 |
| 2016/4/20 | 10.60 | 2.60 | 5.12 | 1.90 | 0.11 | 0.44 | 1.28 | 1.54 | 0.21 | 0.24 | 0.10 |
| 2016/4/21 | 37.50 | 1.44 | 2.41 | 1.38 | 0.12 | 0.28 | 0.59 | 0.72 | 0.13 | 0.26 | 0.14 |
| 2016/4/24 | 8.80 | 2.42 | 4.67 | 2.20 | 0.08 | 0.55 | 1.07 | 1.37 | 0.20 | 0.68 | 0.36 |
| 2016/4/27 | 8.00 | 1.96 | 3.47 | 1.30 | 0.10 | 0.59 | 0.67 | 0.88 | 0.26 | 0.36 | 0.11 |
| 2016/4/28 | 30.50 | 0.97 | 1.24 | 0.51 | 0.07 | 0.15 | 0.22 | 0.27 | 0.11 | 0.16 | 0.00 |
| 2016/4/29 | 32.20 | 3.37 | 5.79 | 2.66 | 0.08 | 0.77 | 1.62 | 1.05 | 0.31 | 0.67 | 0.26 |
| 2016/4/30 | 4.80 | 2.84 | 5.14 | 2.69 | 0.06 | 0.94 | 0.94 | 1.41 | 0.46 | 0.57 | 0.34 |
| 2016/5/1 | 6.40 | 3.60 | 6.25 | 4.14 | 0.08 | 0.92 | 1.42 | 1.63 | 0.43 | 0.79 | 0.43 |
| 2016/5/4 | 35.20 | 2.27 | 3.39 | 2.20 | 0.09 | 0.33 | 1.36 | 0.67 | 0.12 | 0.19 | 0.19 |
| 2016/5/5 | 30.60 | 1.23 | 1.80 | 1.13 | 0.08 | 0.18 | 0.54 | 0.40 | 0.11 | 0.19 | 0.15 |
| 2016/5/6 | 9.60 | 0.77 | 0.92 | 0.75 | 0.09 | 0.25 | 0.01 | 0.48 | 0.14 | 0.12 | 0.00 |
| 2016/5/7 | 33.50 | 1.15 | 2.10 | 0.98 | 0.07 | 0.24 | 0.47 | 0.55 | 0.16 | 0.11 | 0.11 |
| 2016/5/8 | 29.30 | 1.68 | 2.79 | 1.69 | 0.05 | 0.33 | 0.77 | 0.67 | 0.13 | 0.18 | 0.13 |
| 2016/5/11 | 8.80 | 2.32 | 3.94 | 2.38 | 0.10 | 0.43 | 1.32 | 0.64 | 0.15 | 0.32 | 0.21 |
| 2016/5/13 | 8.70 | 0.71 | 1.05 | 0.68 | 0.09 | 0.14 | 0.12 | 0.53 | 0.08 | 0.09 | 0.08 |
| 2016/5/15 | 8.40 | 0.82 | 1.12 | 0.58 | 0.28 | 0.28 | 0.07 | 0.53 | 0.15 | 0.21 | 0.08 |
| 2016/5/18 | 33.70 | 3.20 | 5.80 | 2.88 | 0.06 | 0.50 | 1.60 | 1.11 | 0.19 | 0.31 | 0.23 |
| 2016/5/19 | 42.80 | 2.13 | 2.43 | 1.46 | 0.11 | 0.30 | 0.69 | 0.48 | 0.12 | 0.20 | 0.12 |
| 2016/5/25 | 32.10 | 1.38 | 2.33 | 0.61 | 0.07 | 0.19 | 0.52 | 0.41 | 0.12 | 0.09 | 0.09 |
| 2016/5/29 | 28.80 | 1.52 | 2.35 | 1.14 | 0.10 | 0.38 | 0.46 | 0.24 | 0.21 | 0.24 | 0.16 |
| 2016/6/3 | 35.20 | 0.44 | 0.71 | 0.26 | 0.04 | 0.06 | 0.10 | 0.22 | 0.06 | 0.12 | 0.00 |
| 2016/6/4 | 35.50 | 0.69 | 1.23 | 0.41 | 0.10 | 0.19 | 0.01 | 0.83 | 0.07 | 0.02 | 0.00 |
| 2016/6/7 | 33.60 | 2.88 | 4.56 | 1.92 | 0.20 | 0.44 | 1.19 | 1.87 | 0.22 | 0.16 | 0.16 |
| 2016/6/8 | 31.70 | 2.07 | 3.65 | 1.26 | 0.06 | 0.35 | 0.83 | 1.06 | 0.18 | 0.16 | 0.10 |
| 2016/6/9 | 15.70 | 2.05 | 3.30 | 1.17 | 0.06 | 0.59 | 0.35 | 0.99 | 0.22 | 0.49 | 0.11 |
| 2016/6/10 | 9.60 | 2.16 | 2.82 | 1.09 | 0.04 | 0.43 | 0.35 | 0.70 | 0.08 | 0.25 | 0.11 |
| 2016/6/11 | 32.60 | 1.51 | 2.50 | 0.77 | 0.04 | 0.33 | 0.32 | 0.71 | 0.21 | 0.14 | 0.10 |
| 2016/6/12 | 36.70 | 0.96 | 1.38 | 0.79 | 0.08 | 0.17 | 0.31 | 0.28 | 0.09 | 0.10 | 0.08 |
| 2016/6/13 | 36.60 | 0.89 | 1.46 | 0.36 | 0.10 | 0.13 | 0.25 | 0.27 | 0.10 | 0.09 | 0.08 |
| 2016/6/15 | 44.80 | 0.40 | 0.63 | 0.39 | 0.04 | 0.07 | 0.17 | 0.16 | 0.06 | 0.06 | 0.09 |
| 2016/6/27 | 8.90 | 4.20 | 3.07 | 3.73 | 0.71 | 1.92 | 1.71 | 1.90 | 0.77 | 0.70 | 0.26 |
| 2016/6/30 | 15.70 | 1.50 | 1.07 | 0.58 | 0.07 | 0.33 | 1.10 | 0.41 | 0.06 | 0.27 | 0.09 |
| 2016/7/2 | 32.50 | 1.11 | 1.13 | 0.90 | 0.16 | 0.39 | 0.24 | 0.68 | 0.19 | 0.22 | 0.09 |
| 2016/7/3 | 29.70 | 0.82 | 1.15 | 0.60 | 0.09 | 0.23 | 0.09 | 0.69 | 0.14 | 0.10 | 0.09 |
| 2016/7/4 | 17.50 | 1.28 | 1.44 | 1.25 | 0.28 | 0.75 | 0.00 | 1.08 | 0.33 | 0.46 | 0.18 |
| 2016/7/5 | 33.60 | 0.81 | 1.74 | 0.33 | 0.05 | 0.26 | 0.21 | 0.35 | 0.14 | 0.21 | 0.09 |
| 2016/7/6 | 33.40 | 1.90 | 2.70 | 1.10 | 0.18 | 0.57 | 0.27 | 1.54 | 0.28 | 0.20 | 0.10 |
| 2016/7/16 | 19.20 | 1.46 | 1.49 | 1.00 | 0.21 | 0.54 | 0.14 | 1.10 | 0.24 | 0.20 | 0.10 |
| 2016/7/17 | 17.10 | 1.00 | 1.14 | 0.70 | 0.17 | 0.42 | 0.00 | 0.61 | 0.21 | 0.19 | 0.07 |
| 2016/8/2 | 40.00 | 0.97 | 0.89 | 0.78 | 0.18 | 0.35 | 0.23 | 0.50 | 0.15 | 0.15 | 0.00 |
| 2016/8/3 | 42.00 | 1.29 | 1.76 | 1.06 | 0.18 | 0.71 | 0.60 | 0.61 | 0.16 | 0.31 | 0.12 |
| 2016/8/4 | 7.80 | 1.39 | 1.75 | 0.20 | 0.04 | 0.23 | 0.85 | 0.38 | 0.10 | 0.53 | 0.35 |
| 2016/8/7 | 33.70 | 1.68 | 2.46 | 1.11 | 0.26 | 0.38 | 0.60 | 0.80 | 0.26 | 0.22 | 0.00 |
| 2016/8/10 | 37.00 | 1.89 | 3.10 | 0.79 | 0.05 | 0.40 | 0.16 | 1.15 | 0.21 | 0.24 | 0.17 |
| 2016/8/11 | 37.50 | 2.04 | 3.97 | 1.36 | 0.09 | 0.20 | 0.75 | 0.65 | 0.08 | 0.11 | 0.14 |
| 2016/8/14 | 8.80 | 2.25 | 3.92 | 0.90 | 0.21 | 0.43 | 0.23 | 1.45 | 0.22 | 0.17 | 0.10 |
| 2016/8/25 | 35.20 | 3.68 | 5.94 | 1.42 | 0.24 | 0.56 | 1.07 | 1.45 | 0.33 | 0.30 | 0.22 |
| 2016/10/18 | 33.20 | 1.21 | 1.40 | 0.53 | 0.19 | 0.36 | 0.31 | 0.57 | 0.12 | 0.31 | 0.14 |
| 2016/10/19 | 33.60 | 2.02 | 2.64 | 0.84 | 0.33 | 0.60 | 0.00 | 0.96 | 0.22 | 0.57 | 0.76 |
| 2016/10/24 | 17.60 | 1.92 | 3.25 | 1.28 | 0.17 | 1.35 | 0.52 | 1.38 | 0.60 | 0.63 | 0.16 |
| 2016/10/25 | 11.20 | 4.94 | 4.67 | 4.30 | 1.03 | 2.93 | 0.00 | 2.76 | 1.54 | 1.52 | 0.16 |
| 2016/11/1 | 17.60 | 1.10 | 1.69 | 0.67 | 0.20 | 0.61 | 0.10 | 0.61 | 0.36 | 0.27 | 0.10 |
| 2016/11/10 | 32.10 | 1.17 | 2.19 | 0.38 | 0.19 | 0.39 | 0.12 | 0.80 | 0.22 | 0.18 | 0.13 |
| 2016/11/11 | 19.20 | 1.81 | 3.46 | 0.88 | 0.16 | 0.72 | 0.26 | 0.79 | 0.30 | 0.36 | 0.27 |
| 2016/11/12 | 34.10 | 2.53 | 4.63 | 1.18 | 0.16 | 0.53 | 0.46 | 0.99 | 0.18 | 0.31 | 0.14 |
| 2016/11/13 | 35.20 | 2.13 | 2.83 | 1.04 | 0.19 | 0.60 | 0.35 | 0.79 | 0.16 | 0.29 | 0.13 |
| 2016/11/14 | 23.40 | 1.93 | 3.04 | 0.93 | 0.32 | 0.56 | 0.55 | 1.08 | 0.25 | 0.28 | 0.00 |
| 2016/11/21 | 15.70 | 1.12 | 1.92 | 0.78 | 0.27 | 0.41 | 0.34 | 0.83 | 0.14 | 0.15 | 0.09 |
| 2016/11/24 | 34.80 | 0.65 | 0.58 | 0.50 | 0.24 | 0.27 | 0.11 | 0.54 | 0.10 | 0.13 | 0.00 |
| 2016/11/25 | 8.00 | 1.18 | 1.91 | 1.14 | 0.31 | 0.35 | 0.22 | 1.00 | 0.17 | 0.26 | 0.12 |
| 2016/11/27 | 32.00 | 0.68 | 0.89 | 0.36 | 0.21 | 0.27 | 0.13 | 0.45 | 0.14 | 0.13 | 0.00 |
| 2016/12/12 | 8.20 | 6.93 | 9.12 | 5.29 | 0.24 | 3.50 | 0.69 | 8.96 | 0.66 | 1.25 | 0.26 |
| 2016/12/19 | 30.20 | 3.01 | 4.57 | 2.64 | 0.28 | 0.98 | 1.17 | 1.14 | 0.23 | 0.56 | 0.20 |
| 2016/12/20 | 39.30 | 2.21 | 3.28 | 2.49 | 0.31 | 0.93 | 1.06 | 0.90 | 0.18 | 0.56 | 0.54 |
| 2016/12/21 | 24.00 | 2.66 | 4.38 | 2.57 | 0.15 | 0.63 | 1.29 | 0.81 | 0.19 | 0.39 | 0.19 |
| 2016/12/24 | 16.20 | 4.95 | 8.25 | 5.52 | 0.12 | 0.71 | 2.20 | 1.34 | 0.23 | 0.35 | 0.28 |
| 2016/12/25 | 33.90 | 2.97 | 4.68 | 2.76 | 0.18 | 0.55 | 1.30 | 0.67 | 0.15 | 0.28 | 0.26 |

**(c) Longyinlong primary school**

| Date | Rain | Ec | SO_4_^2-^ | NO_3_^-^ | F^-^ | Cl^-^ | NH_4_^+^ | Ca^2+^ | Mg^2+^ | Na^+^ | K^+^ |
| --- | --- | --- | --- | --- | --- | --- | --- | --- | --- | --- | --- |
|  | (mm) | (ms/m) | (mg/L) | (mg/L) | (mg/L) | (mg/L) | (mg/L) | (mg/L) | (mg/L) | (mg/L) | (mg/L) |
| 2016/1/5 | 11.40 | 6.17 | 11.20 | 2.22 | 0.23 | 4.51 | 1.57 | 2.59 | 0.65 | 3.84 | 1.26 |
| 2016/1/8 | 16.20 | 3.92 | 5.36 | 3.08 | 0.10 | 0.69 | 1.15 | 1.70 | 0.11 | 0.26 | 0.22 |
| 2016/1/9 | 15.40 | 3.20 | 3.94 | 2.05 | 0.11 | 0.42 | 0.83 | 0.94 | 0.04 | 0.14 | 0.17 |
| 2016/1/14 | 7.20 | 2.15 | 3.07 | 1.26 | 0.15 | 0.37 | 0.45 | 0.95 | 0.09 | 0.26 | 0.19 |
| 2016/1/15 | 10.40 | 1.50 | 2.44 | 1.04 | 0.04 | 0.23 | 0.28 | 0.75 | 0.05 | 0.19 | 0.16 |
| 2016/1/28 | 6.40 | 2.10 | 3.22 | 0.71 | 0.12 | 1.21 | 0.32 | 1.32 | 0.26 | 0.52 | 0.32 |
| 2016/1/31 | 14.00 | 1.04 | 1.39 | 0.89 | 0.04 | 0.28 | 0.28 | 0.54 | 0.07 | 0.17 | 0.12 |
| 2016/2/1 | 15.00 | 1.48 | 2.11 | 1.45 | 0.04 | 0.27 | 0.47 | 0.75 | 0.07 | 0.12 | 0.19 |
| 2016/3/9 | 20.70 | 2.97 | 5.02 | 3.01 | 0.10 | 0.65 | 1.52 | 1.36 | 0.28 | 0.35 | 0.36 |
| 2016/3/10 | 35.00 | 3.68 | 6.75 | 2.86 | 0.09 | 0.77 | 1.35 | 1.74 | 0.39 | 0.32 | 0.33 |
| 2016/3/17 | 25.00 | 4.09 | 6.94 | 2.59 | 0.14 | 1.46 | 1.28 | 2.30 | 0.45 | 0.94 | 0.31 |
| 2016/3/20 | 50.60 | 4.49 | 6.45 | 3.18 | 0.13 | 2.34 | 0.74 | 3.21 | 0.39 | 0.99 | 0.30 |
| 2016/3/21 | 33.50 | 3.91 | 6.34 | 3.17 | 0.21 | 1.48 | 1.24 | 2.17 | 0.39 | 0.86 | 0.24 |
| 2016/3/22 | 44.20 | 1.76 | 2.22 | 1.26 | 0.23 | 0.21 | 0.63 | 0.40 | 0.10 | 0.12 | 0.11 |
| 2016/4/3 | 60.20 | 0.68 | 0.64 | 0.45 | 0.18 | 0.13 | 0.10 | 0.29 | 0.08 | 0.14 | 0.11 |
| 2016/4/5 | 10.50 | 4.00 | 5.40 | 4.20 | 0.13 | 1.09 | 2.16 | 1.49 | 0.25 | 0.63 | 0.38 |
| 2016/4/8 | 36.50 | 1.87 | 3.13 | 1.41 | 0.09 | 0.24 | 0.76 | 0.45 | 0.11 | 0.32 | 0.13 |
| 2016/4/9 | 28.80 | 2.74 | 4.44 | 2.77 | 0.08 | 0.59 | 1.55 | 0.65 | 0.14 | 0.39 | 0.26 |
| 2016/4/10 | 36.60 | 1.23 | 1.57 | 0.82 | 0.06 | 0.11 | 0.40 | 0.23 | 0.07 | 0.19 | 0.10 |
| 2016/4/11 | 36.70 | 1.06 | 1.47 | 1.00 | 0.13 | 0.14 | 0.41 | 0.44 | 0.08 | 0.20 | 0.11 |
| 2016/4/12 | 9.80 | 2.93 | 5.06 | 2.80 | 0.17 | 0.61 | 1.24 | 1.63 | 0.18 | 0.58 | 0.30 |
| 2016/4/16 | 28.80 | 2.34 | 4.77 | 2.37 | 0.10 | 0.54 | 1.59 | 1.34 | 0.17 | 0.38 | 0.23 |
| 2016/4/17 | 35.80 | 2.89 | 2.78 | 2.25 | 0.06 | 0.35 | 0.97 | 0.33 | 0.11 | 0.20 | 0.19 |
| 2016/4/19 | 12.80 | 1.58 | 2.90 | 1.56 | 0.11 | 0.43 | 0.82 | 0.78 | 0.15 | 0.28 | 0.19 |
| 2016/4/20 | 9.60 | 2.88 | 5.92 | 2.73 | 0.19 | 0.53 | 1.21 | 3.01 | 0.21 | 0.37 | 0.19 |
| 2016/4/21 | 38.70 | 1.52 | 2.00 | 1.17 | 0.10 | 0.18 | 0.55 | 0.56 | 0.08 | 0.16 | 0.13 |
| 2016/4/24 | 9.50 | 2.33 | 4.03 | 1.36 | 0.13 | 0.39 | 0.72 | 1.07 | 0.23 | 0.28 | 0.11 |
| 2016/4/27 | 8.60 | 2.49 | 3.76 | 1.56 | 0.25 | 0.81 | 0.87 | 1.14 | 0.31 | 0.59 | 0.15 |
| 2016/4/28 | 33.10 | 1.12 | 1.14 | 0.50 | 0.16 | 0.12 | 0.18 | 0.25 | 0.06 | 0.24 | 0.10 |
| 2016/4/29 | 32.20 | 3.11 | 4.74 | 2.44 | 0.18 | 0.55 | 1.44 | 0.84 | 0.18 | 0.59 | 0.31 |
| 2016/5/4 | 38.10 | 2.38 | 3.40 | 2.00 | 0.10 | 0.34 | 1.24 | 0.60 | 0.12 | 0.27 | 0.19 |
| 2016/5/5 | 31.30 | 1.22 | 1.74 | 0.97 | 0.04 | 0.18 | 0.45 | 0.34 | 0.09 | 0.16 | 0.12 |
| 2016/5/7 | 33.90 | 1.50 | 2.56 | 1.36 | 0.05 | 0.28 | 0.77 | 0.51 | 0.09 | 0.17 | 0.15 |
| 2016/5/8 | 26.90 | 1.88 | 2.57 | 1.55 | 0.12 | 0.30 | 0.74 | 0.37 | 0.10 | 0.14 | 0.13 |
| 2016/5/11 | 32.10 | 2.75 | 4.37 | 2.74 | 0.13 | 0.49 | 1.31 | 0.65 | 0.16 | 0.33 | 0.23 |
| 2016/5/13 | 32.20 | 0.75 | 0.96 | 0.37 | 0.04 | 0.12 | 0.05 | 0.23 | 0.09 | 0.15 | 0.09 |
| 2016/5/15 | 32.00 | 0.77 | 1.14 | 0.52 | 0.05 | 0.20 | 0.20 | 0.45 | 0.09 | 0.10 | 0.08 |
| 2016/5/18 | 33.20 | 3.27 | 6.06 | 3.18 | 0.16 | 0.53 | 1.78 | 1.22 | 0.22 | 0.33 | 0.24 |
| 2016/5/19 | 44.30 | 1.44 | 2.24 | 1.25 | 0.08 | 0.30 | 0.60 | 0.42 | 0.13 | 0.22 | 0.12 |
| 2016/5/25 | 32.50 | 1.03 | 1.84 | 0.55 | 0.07 | 0.16 | 0.34 | 0.39 | 0.07 | 0.06 | 0.08 |
| 2016/5/29 | 17.60 | 1.89 | 2.86 | 0.98 | 0.19 | 0.50 | 0.51 | 0.26 | 0.24 | 0.26 | 0.14 |
| 2016/6/3 | 35.30 | 0.36 | 0.56 | 0.30 | 0.04 | 0.09 | 0.06 | 0.21 | 0.06 | 0.07 | 0.10 |
| 2016/6/4 | 35.00 | 0.59 | 0.84 | 0.30 | 0.05 | 0.14 | 0.09 | 0.27 | 0.08 | 0.09 | 0.08 |
| 2016/6/7 | 16.00 | 2.72 | 4.51 | 1.52 | 0.70 | 0.64 | 0.90 | 2.12 | 0.07 | 0.30 | 0.16 |
| 2016/6/8 | 16.00 | 2.16 | 3.59 | 1.80 | 0.20 | 0.47 | 0.62 | 1.91 | 0.17 | 0.21 | 0.13 |
| 2016/6/9 | 11.20 | 1.88 | 3.60 | 1.90 | 0.13 | 0.36 | 0.59 | 2.00 | 0.13 | 0.21 | 0.11 |
| 2016/6/10 | 9.60 | 2.02 | 2.92 | 0.89 | 0.14 | 0.55 | 0.10 | 0.97 | 0.29 | 0.31 | 0.10 |
| 2016/6/11 | 31.80 | 1.10 | 1.88 | 0.69 | 0.04 | 0.15 | 0.40 | 0.42 | 0.09 | 0.11 | 0.10 |
| 2016/6/12 | 37.40 | 0.77 | 1.19 | 0.34 | 0.10 | 0.12 | 0.26 | 0.29 | 0.09 | 0.08 | 0.08 |
| 2016/6/13 | 36.50 | 0.84 | 1.11 | 0.74 | 0.08 | 0.17 | 0.30 | 0.26 | 0.07 | 0.11 | 0.11 |
| 2016/6/15 | 44.50 | 0.35 | 0.59 | 0.36 | 0.03 | 0.08 | 0.13 | 0.12 | 0.06 | 0.07 | 0.13 |
| 2016/6/27 | 8.10 | 3.78 | 3.12 | 2.95 | 0.58 | 1.52 | 2.57 | 0.97 | 0.27 | 0.68 | 0.27 |
| 2016/6/30 | 16.00 | 2.03 | 1.48 | 1.76 | 0.38 | 0.86 | 0.54 | 1.05 | 0.38 | 0.46 | 0.16 |
| 2016/7/2 | 30.60 | 0.95 | 1.04 | 0.78 | 0.16 | 0.35 | 0.27 | 0.67 | 0.13 | 0.20 | 0.10 |
| 2016/7/3 | 33.70 | 1.08 | 1.28 | 0.90 | 0.10 | 0.37 | 0.36 | 0.60 | 0.14 | 0.18 | 0.10 |
| 2016/7/5 | 33.70 | 0.79 | 0.96 | 0.38 | 0.08 | 0.23 | 0.14 | 0.50 | 0.11 | 0.14 | 0.07 |
| 2016/7/6 | 33.60 | 1.63 | 1.90 | 1.19 | 0.13 | 0.30 | 0.16 | 1.04 | 0.10 | 0.13 | 0.08 |
| 2016/7/16 | 9.60 | 2.55 | 3.19 | 1.37 | 0.31 | 1.03 | 0.41 | 1.38 | 0.24 | 0.60 | 0.14 |
| 2016/11/14 | 21.30 | 6.48 | 9.83 | 3.37 | 0.24 | 1.64 | 0.23 | 7.80 | 0.26 | 0.42 | 0.48 |
| 2016/11/21 | 18.20 | 0.98 | 1.30 | 0.49 | 0.32 | 0.35 | 0.16 | 0.92 | 0.08 | 0.14 | 0.11 |
| 2016/11/24 | 35.20 | 0.80 | 0.99 | 0.49 | 0.22 | 0.26 | 0.13 | 0.40 | 0.11 | 0.08 | 0.13 |
| 2016/12/12 | 8.30 | 5.10 | 10.30 | 5.36 | 0.33 | 3.28 | 1.95 | 2.58 | 0.43 | 2.46 | 0.17 |
| 2016/12/19 | 28.80 | 3.27 | 5.32 | 3.39 | 0.24 | 1.15 | 1.57 | 1.79 | 0.19 | 0.64 | 0.20 |
| 2016/12/20 | 38.20 | 2.21 | 3.42 | 2.86 | 0.30 | 0.80 | 1.16 | 0.83 | 0.15 | 0.57 | 0.39 |
| 2016/12/21 | 33.60 | 2.91 | 4.55 | 2.88 | 0.22 | 0.59 | 1.25 | 0.89 | 0.15 | 0.36 | 0.24 |
| 2016/12/25 | 34.00 | 3.63 | 5.20 | 3.54 | 0.89 | 0.45 | 1.46 | 2.37 | 0.14 | 0.25 | 0.28 |
